# A model of digestive tooth corrosion in lizards: experimental tests and taphonomic implications

**DOI:** 10.1038/s41598-021-92326-5

**Published:** 2021-06-18

**Authors:** Krister T. Smith, Orr Comay, Lutz Maul, Fabio Wegmüller, Jean-Marie Le Tensorer, Tamar Dayan

**Affiliations:** 1 Department of Messel Research and Mammalogy, Senckenberg Research Institute, Senckenberganlage 25, 60325 Frankfurt, Germany; 2 Institute for Ecology, Diversity and Evolution, Faculty of Biological Sciences, Goethe University, Max-von-Laue-Strasse 13, 60438 Frankfurt, Germany; 3 School of Zoology and The Steinhardt Museum of Natural History, Tel Aviv University, 69978 Tel Aviv, Israel; 4 Research Station of Quaternary Palaeontology, Senckenberg Research Institute, Am Jakobskirchhof 4, 99423 Weimar, Germany; 5 Institute for Prehistory and Archaeological Science, University of Basel, Spalenring 145, 4055 Basel, Switzerland

**Keywords:** Palaeontology, Archaeology

## Abstract

Corrosion patterns induced by gastric fluids on the skeleton of prey animals may depend on the nature of the corrosive agents (acid, enzymes) as well as on the composition of the hard parts and the soft tissues that surround them. We propose a framework for predicting and interpreting corrosion patterns on lizard teeth, our model system, drawing on the different digestive pathways of avian and non-avian vertebrate predators. We propose that high-acid, low-enzyme systems (embodied by mammalian carnivores) will lead to corrosion of the tooth crowns, whereas low-acid, high-enzyme systems (embodied by owls) will lead to corrosion of the tooth shafts. We test our model experimentally using artificial gastric fluids (with HCl and pepsin) and feeding experiments, and phenomenologically using wild-collected owl pellets with lizard remains. Finding an association between the predictions and the experimental results, we then examine corrosion patterns on nearly 900 fossil lizard jaws. Given an appropriate phylogenetic background, our focus on physiological rather than taxonomic classes of predators allows the extension of the approach into Deep Time.

## Introduction

“Signs and the signs of signs are used only when we are lacking things.”

—Umberto Eco, *The Name of the Rose* (1983, trans.)

Predators play an outsize role in vertebrate taphonomy. By egesting the indigestible hard parts—particularly hydroxyapatite, the mineral phase of bones and teeth, but also carbonate shells—predators help to concentrate them, leading to discrete spatial accumulations of teeth and bones in the fossil record. In most fossil localities, it is only the hard parts of animals that are preserved. Consequently, considerable effort has been devoted to understanding modifications to hard parts caused by their passage through the digestive tract of vertebrate predators^1–5^. However, compared to mammal prey, little attention has been paid to predation on lizards, a group with an even higher species diversity that appears in large numbers in many paleontological and archeological sites spanning more than 150 million years^6^.

Pre-depositional corrosion of hard parts is considered a hallmark of digestive modification in the vertebrate fossil record^3^. The localization of corrosion on a prey skeleton, however, might vary depending on the nature of the corrosive agent (acid, proteases), the behavior and digestive physiology of the predator, the composition of the hard parts, and the soft tissue that surrounds them. In this paper we develop a new conceptual framework for predicting and interpreting digestive corrosion on lizard teeth, our model system. This framework exploits differences in the digestive pathways of avian vs. mammalian predators and serves several purposes. It is a first step in understanding and explaining corrosion phenomena in lizards. Lizards are common faunal components from the Jurassic to Quaternary^7,8^, but their dentition is very different from that of mammals. Hence, previous actuotaphonomic works focusing on micromammals^3,4,9^ are not readily applicable to lizards. Furthermore, any clues that the lizards provide as to the identity of a bone accumulator will help to explain the accumulation as a whole. We focus on physiological rather than taxonomic classes, which allows extension of the framework into Deep Time. We test our predictions on lizard carcasses using artificial gastric fluids and real predators, both captive and wild. Then, we examine corrosion patterns in a variety of Quaternary (Qesem Cave, Hummal) and Paleogene fossil localities. Finally, we discuss digestive strategies of avian and non-avian vertebrate predators in light of the results.

## A model of digestive corrosion on lizard teeth

**Figure 1:**
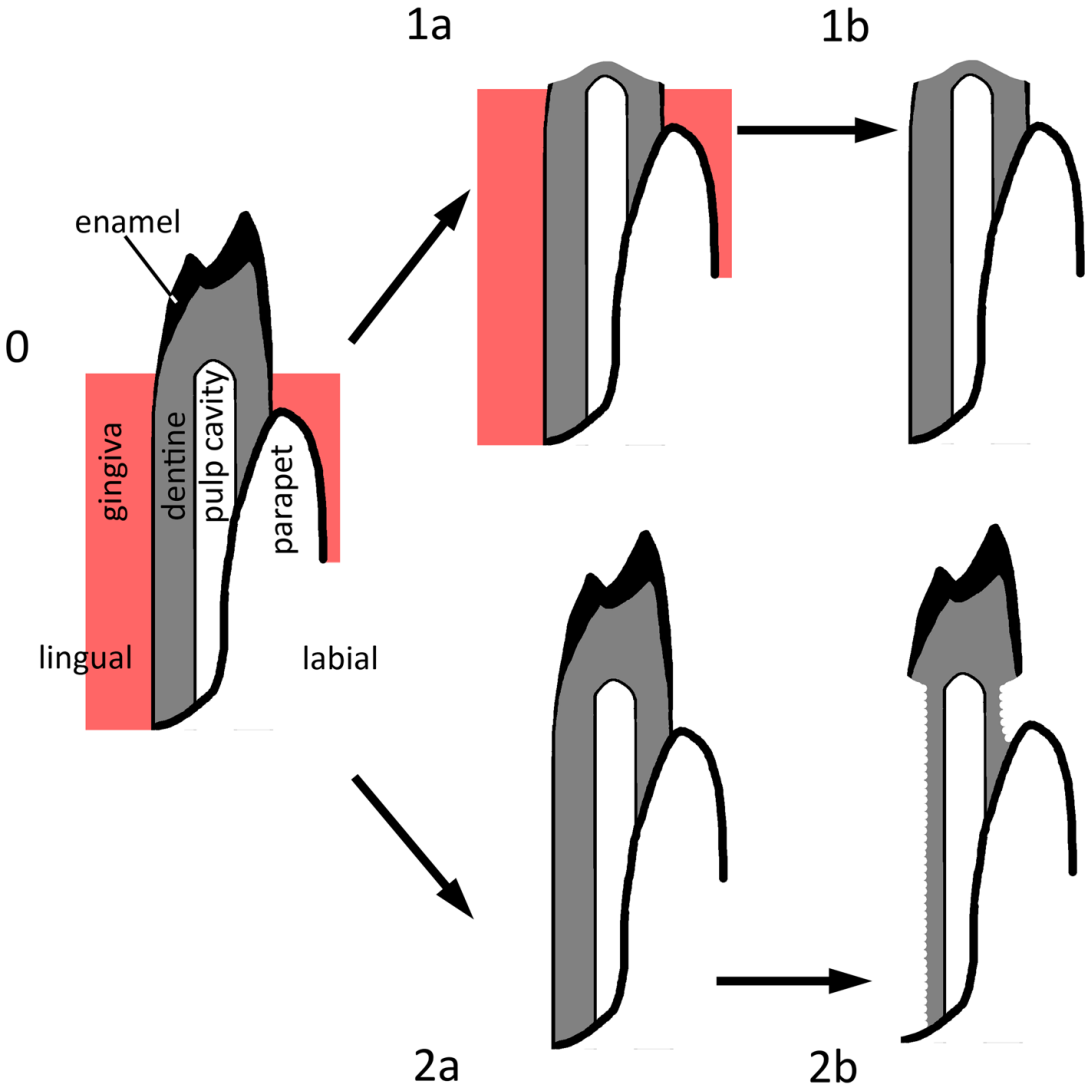
Cartoon depicting an idealized model of digestive corrosion on lizard teeth, shown here in transverse section. In the initial state (0), the crown, largely composed of enamel, is exposed, whereas the shaft, largely composed of dentine, is covered lingually by the gingiva and labially by bone (the parapet of the jaw). In the enteric digesters, the gingiva remains intact while the crown is attacked by hydrochloric acid (**1a**); digestion of soft tissue then occurs in the intestine after corrosion has ceased (**1b**). As a result, the crown of the tooth is destroyed while the shaft is undamaged; this is type 1 corrosion. In the gastric digesters, the gingiva is digested already in the stomach (**2a**). Dentine is susceptible to corrosion by acid and the protease pepsin, so if acidity is low, the shaft of the tooth is corroded while the tip is undamaged (**2b**). This is type 2 corrosion.

Lizards show pleurodont tooth implantation (Fig. 1), where the tooth shafts are attached labially to the lateral wall of the jaw (the parapet), but their lingual surfaces are covered only by the gingiva^10^. The tooth crown, the tip, juts out from the gingiva to interact with food. Thus, the crown is exposed, whereas the shaft (particularly its lingual surface) is covered only by flesh. The kinds of dental tissues also vary from base to tip on the tooth. The vitreous enamel is thick on the crown^11,12^, whereas the shaft comprises a “dentine cone”^11^ (Fig. S1). Some enamel extends thinly down the shaft, but only for a short distance. Enamel in lizards (as in other jawed vertebrates) consists almost entirely of the mineral phase, hydroxyapatite, because the protein matrix is destroyed during maturation^11,12^. In contrast, dentine retains a significant proportion of the protein matrix, principally collagen.

Two major, potentially corrosive substances are present in the stomach of jawed vertebrates, hydrochloric acid and the protease pepsin. Corrosion by HCl, while associated with digestion, is incidental to it. HCl may serve an antiseptic role^13,14^, but its primary digestive role is to lower the pH of the gastric fluid, which is necessary in order to transform the precursor pepsinogen, also secreted by the stomach, into the active form of the enzyme, pepsin^15^. Beyond the stomach, in the intestine, the pH rises toward neutrality.

These corrosive agents—HCl and enzymes—have different effects on dental tissue depending on its composition^16–18^. On the one hand, acid corrodes the mineral phase. The effects of strong acid (pH = 1) on enamel are visible after only minutes^19^. The mineral phase is thought to be stable in basic solutions^20^. Acid will have only a limited effect on the protein phase; in particular, it may denature (unfold) but does not digest collagen^21^. On the other hand, protease enzymes, in particular pepsin, will have no influence on the mineral phase of dental tissue but will digest the collagen that makes up the protein matrix^22^. Thus, enamel will only be affected by HCl, whereas dentine, because it consists of both mineral and protein, should be subject to corrosion by both HCl and pepsin. The experiments of Cummings et al.^23^ using HCl and pepsin confirmed this.

We hypothesize that two end-member physiologies will produce different corrosion patterns on the teeth of pleurodont lizards. In particular, we predict that in high-acid, low-enzyme systems, the crown of a lizard tooth will be much more heavily corroded than the shaft. The reasoning is that if enzymatic activity is low, the gingiva will not be digested strongly in the stomach, so the shafts will not be exposed to enzymes or acid; in contrast, the enamel crowns will be corroded by the acid (Fig. 1, upper sequence). We call this corrosion pattern ‘type 1’. Examined mammalian carnivores generally correspond to this physiology (Supplementary Note). We also predict that in low-acid, high-enzyme systems, the shaft will be more corroded than the crown. The reasoning is that the jaw will quickly be defleshed, uniformly exposing the entire tooth to gastric fluids. The weak acid and enzymes will work in concert to digest the dentine, leaving a well-demarcated step between corroded shaft and enameled crown (Fig. 1, lower sequence). We call this corrosion pattern ‘type 2’. Examined owls generally correspond to this physiology (Supplementary Note). In addition, we predict that high-acid, high-enzyme systems will be extremely corrosive to both crown and shaft, as well (potentially) of the bone that supports the tooth. Examined diurnal raptors generally correspond to this physiology (Supplementary Note). In contrast, we predict that low-acid, low-enzyme systems will have no discernible effect on the teeth.

## Results

### Artificial gastric fluids

Experiments with artificial gastric fluids comprising HCl and pepsin were not conclusive with respect to the predictions (Supplementary Data). In general, artificial gastric fluids tended not to produce extensive degradation of the carcasses, especially as would be expected for the low-acid, high-enzyme solutions most similar to the gastric fluids of owls.

Corrosion approximating the predicted type 1 corrosion (Fig. 1) was commonly observed, whereas type 2 (Fig. 1) corrosion was rare. Nevertheless, cases of type 2 corrosion were all concentrated in the low-acid, high-enzyme part of parameter space (Supplementary Fig. S3), as predicted by the model. Statistically speaking, high-acid, low-enzyme and low-acid, high-enzyme solutions were not significantly associated with a greater incidence of crown vs. shaft corrosion according to Fisher’s exact test. However, multiple logistic regression of pH, pepsin concentration and duration on all treatments (*N* = 20) producing approximations of type 1 and type 2 corrosion (Supplementary Fig. S3) yields positive (favoring type 2 over type 1) regression coefficients with significant *P*-values for pH (*P* = 0.00028) and pepsin concentration (*P* = 0.024), as predicted by the model (Supplementary Data).

### Feeding experiments: Ferret

**Figure 2:**
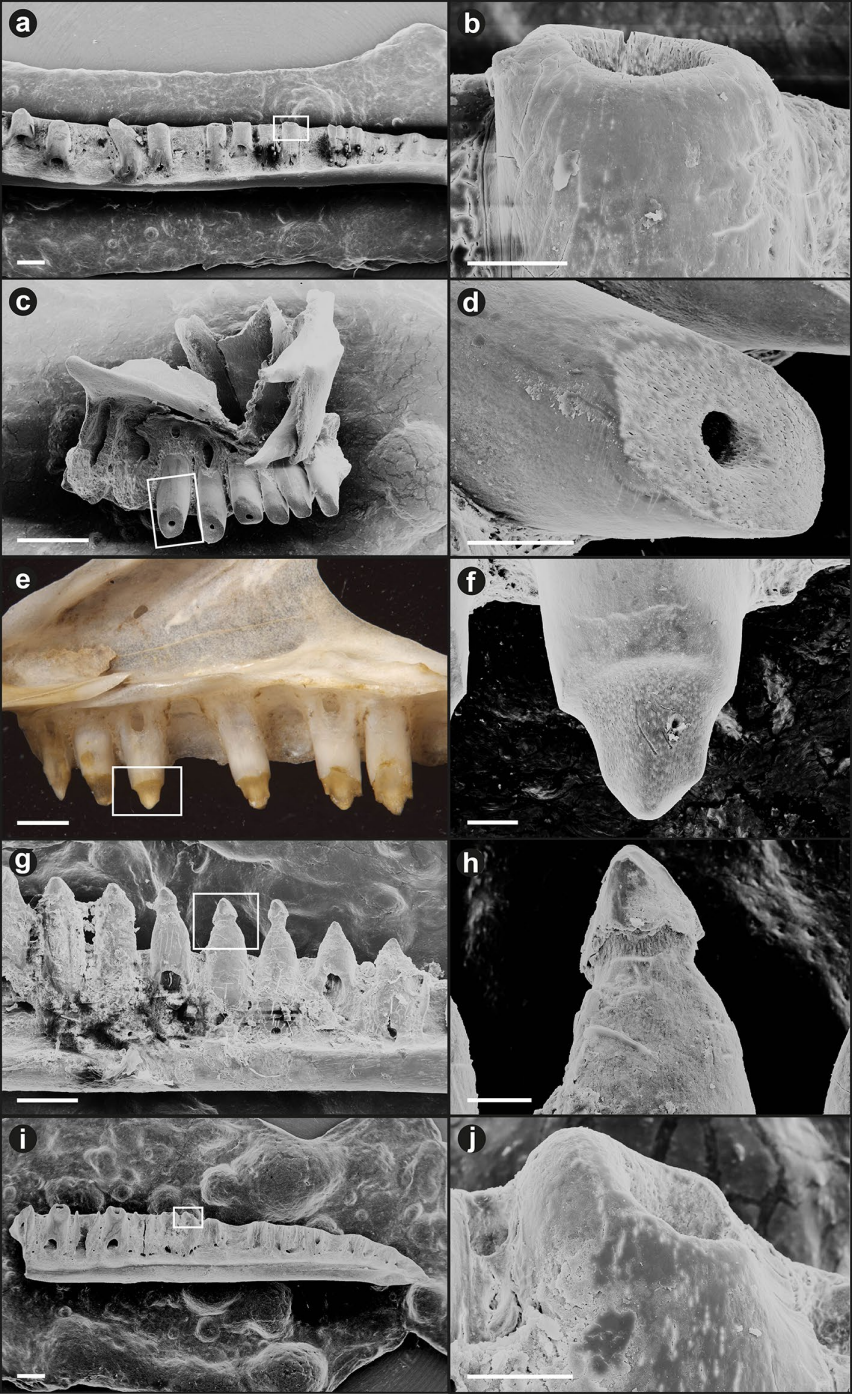
Corrosion damage to teeth of Green Anoles (*Anolis carolinensis*) ingested by Ferrets (*Mustela putorius*). (**a**–**b**) whole lower jaw and close-up of tooth, where the crown is completely destroyed and the shaft is damaged, exposing small pits; (**c**–**d**) whole premaxilla and close-up of tooth, where the crown is completely destroyed and the shaft is not damaged; (**e**–**f**) whole upper jaw and close-up of tooth, where the crown is decalcified (note pin-prick) but the shaft is not damaged; (**g**–**h**) tooth series from and close-up of tooth crown from lower jaw, showing antero-posterior gradient and ’neck’ corrosion; (**i**–**j**) whole lower jaw, showing antero-posterior gradient, and close-up of tooth. Scale bars: left panels 0.5 mm, right panels 100 µm.

The Ferrets are expected to have high-acid, low-enzyme gastric fluids (Supplementary Note). The bones were considerably fragmented, because the Ferrets chewed the carcasses thoroughly. We focused on the middle region of the maxilla and dentary, with a minimum number of individuals (MNI) of 10 (Table 1). Twenty-one (of 66) middle maxilla and 38 (of 66) middle dentary fragments were recovered, for a minimum recovery rate of 32% and 58%, respectively. The vast majority of these showed at least some corrosive damage to the teeth (Table 1; Fig. 2). Occasionally, the dentition was almost completely destroyed; the extent to which previous breakage might have contributed to destruction of the teeth is uncertain, but similar results were obtained in our laboratory experiment under high-acid, low-enzyme conditions, suggesting that corrosion is the overriding factor here. Only 14% showed no corrosion on either crown or shaft. In 88% the damage to the crown was greater than the damage to the shaft (Table 1).

**Table 1:** Summary of corrosion damage to teeth of Green Anoles (*Anolis carolinensis*) ingested by Ferrets (*Mustela putorius*).

	*N*	*n* showing corrosion (C) to crown	*n* showing corrosion (C) to shaft	C(crown) > C(shaft)	C(shaft) > C(crown)
**Cage 1 (2 Ferrets, 18 lizard heads):**					
L maxilla	10	7	9	7	3
L dentary	11	9	3	7	0
R maxilla	5	4	3	3	1
R dentary	13	9	7	9	1
**Cage 2 (2 Ferrets, 15 lizard heads):**					
L maxilla	3	3	3	3	0
L dentary	7	4	2	1	0
R maxilla	3	2	2	2	0
R dentary	7	5	2	3	0
**TOTALS**	59	43	31	35	5

A total of 48 jaws showed corrosion to crown or shaft, whereas 8 showed no corrosion. The remaining 3 jaws could not be cleaned sufficiently to assess.

In a high proportion of specimens (Table 1) the crowns were destroyed and shafts preserved on most teeth (Fig. 2a, b), or crowns decalcified (Fig. 2e, f), with only the protein matrix remaining. In some cases the shafts of the teeth were corroded as well (Fig. 2i, j), whereas in others they were uncorroded and the crowns either appeared sheared off (Fig. 2c, d) or were decalcified. Where sheared off, the homogeneous character of the damage suggests it is corrosion whose extent was controlled by surrounding soft tissues (the gingiva). We consider the alternative—that the tooth crowns were first broken before they were corroded—to be unlikely, as it would require a series of adjacent teeth to break in precisely the same way despite the fact that the series is longer than individual cusps of Ferret teeth.

Within a single jaw—where these are substantially complete—it is common for the degree of corrosion to decrease from anterior to posterior (Fig. 2i, j), since corrosive fluids must enter through the mouth, and the oral opening is considerably larger than the esophageal opening.

There appear to be two distinct mechanisms of crown destruction. The first mechanism is decalcification (Fig. 2e, f), whereby apical to the gum-line the tooth is distinctly restricted in girth, the enamel apparently missing, and the dentine represented largely by a brownish protein matrix. As noted above, this matrix would decay in the diagenetic environment. The second mechanism is ‘popping off’, particularly well shown by a specimen with a corrosion gradient (Fig. 2g, h). Here, the posterior teeth are less corroded. Moving from posterior to anterior, the neck of the tooth between the crown and the shaft becomes increasingly constricted by corrosion; ultimately, it appears that the neck became so thin that the remaining tip of the crown broke off without the enamel of that portion—or the underlying dentine—ever having been corroded. A total of 5 specimens show this intermediate condition, in which corrosion has caused a strong neck to develop on the tooth just above the former level of the gum-line. For ease of reference, we call this condition ‘neck corrosion’. As will be shown later, it can be related to both high-acid, low-enzyme (as here) and low-acid, high-enzyme physiologies.

### Feeding experiments: Honey Badger

Like the Ferrets, the Honey Badger chewed its prey thoroughly before swallowing, starting with the head. No teeth or jaws were found in the Honey Badger’s scat, and only some green undigested remains and a few small bone fragments testified that the 11 anoles were eaten at all. On the other hand, chick bones were abundant. The reason for the differential treatments of anole and chick bones is not certain, but the much smaller body size of the anoles probably plays a role.

### Feeding experiments: Kestrel

The Kestrels moved the carcasses around their cage and ate the flesh around the bones, but no mandibles nor maxillae were found in their pellets, and the only bones found were those of a single anole leg. As our study concerns tooth corrosion, this experiment did not produce analyzable results, and it will not be further discussed.

### Feeding experiments: Barn Owl

The Barn Owls are expected to have low-acid, high-enzyme gastric fluids (Supplementary Note). The Barn Owls swallowed their prey whole, without tearing it apart first. Total damage to the teeth was much less than in the Ferret experiment. Consistent with previous observations of owl vs. mammal feeding, the lizard jaws in this experiment were for the most part recovered whole. Corrosion is in most cases light. In a number of cases (19% for individual 1, 45% for individual 2) corrosion was absent entirely or could not clearly be identified. These values—weighted average 25%—exceed the 14% uncorroded specimens found with the Ferrets.

**Figure 3:**
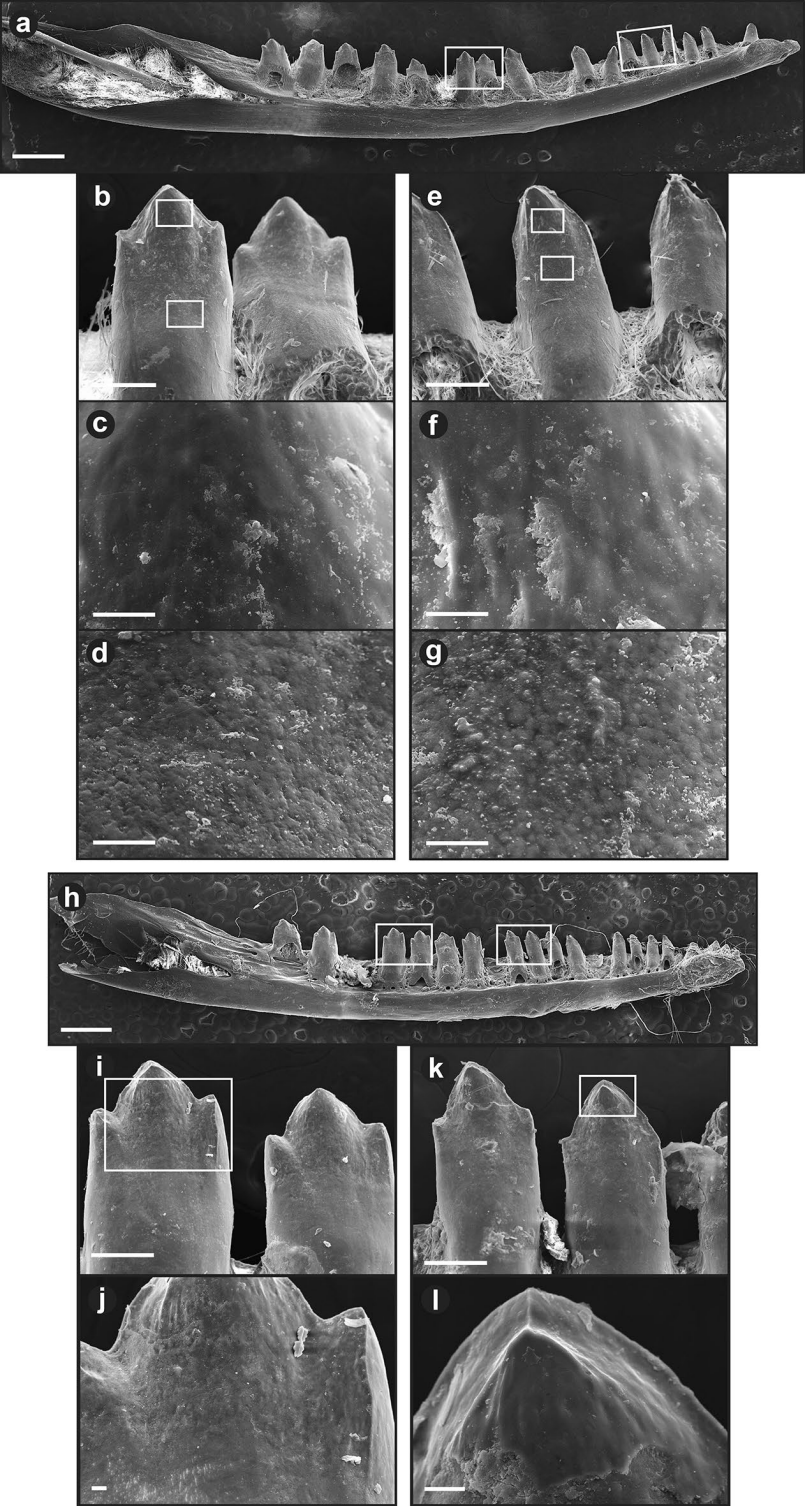
Corrosion damage to teeth of Green Anoles (*Anolis carolinensis*) ingested by Barn Owls (*Tyto alba*): (**a**–**g**) whole lower jaw, whole teeth and close-ups of crown and shaft of specimen #H4, where corrosion is confined to the shaft; (**h**–**l**) whole lower jaw, whole teeth and close-ups of crown and shaft of specimen #F2, where corrosion has proceeded onto the crown, leaving on the front teeth a discrete step. Scale bars: (**a**) and (**h**), 1 mm; (**b**, **e**, **i**) and (**k**), 200 µm; (**c**–**d**, **f**–**g**, **j**), and (**l**), 20 µm.

The great majority of specimens showed corrosion on the shaft, and corrosive damage to the shaft exceeding corrosive damage to the crown was observed in many more specimens than the reverse (Table 2). Observed corrosion was most commonly on the middle to upper shaft, i.e., from the level of the jaw parapet to the base of the crown (Fig. 3). Because this area lies beneath the gingiva on an intact specimen, the gingiva was probably digested to allow easy access of corrosive fluids to the shaft. Corrosion typically—although not always (#H22, H23)—did not extend to the very base of the shaft.

**Table 2:** Summary of corrosion damage to teeth of Green Anoles (*Anolis carolinensis*) ingested by Barn Owls (*Tyto alba*).

	*N*	*n* showing corrosion (C) to crown	*n* showing Corrosion (C) to shaft	C(crown) > C(shaft)	C(shaft) > C(crown)
Individual 1	37	21	28	1	12
Individual 2	11	1	5	0	4
TOTALS	48	22	33	1	16

Because jaws were generally whole, and there was frequently variation in degree of corrosion from anterior to posterior, the counts are based on the middle teeth (10–15) of the jaw. The number of uncorroded specimens cannot be derived by subtracting from the number of jaws (*N*), because some specimens could not be fully evaluated because of adhering matter (dirt, hair).

Frequently there was no clearly demarcated boundary between corroded and uncorroded surfaces (Fig. 3a–g). Rather, the degree of corrosion appeared simply to diminish apically. However, in 9 jaws (#H11, H12, H14, H22, H23, F2, K4, K7, K11) the corroded portion (typically upper shaft) was well-demarcated and separated by a distinct line and often a strong step (Fig. 3h–l) from the uncorroded portion (typically the crown). In a single specimen, the tip of the crown of one tooth was so deeply separated from the shaft by a constriction that the tooth approximated ’neck’ corrosion (see above).

A trend of increasing corrosion intensity is commonly present on specimens from back to front. In some cases it involves only a slight increase in the vertical extent of corrosion (e.g., #H15), which increases towards the front. In other cases, the extent of corrosion is considerably greater on anterior teeth, where the enamel of the crowns may be entirely lost or (in 10 cases) even the entire crown or tooth destroyed. In such cases, some anterior teeth show a type 1 corrosion pattern (Fig. 1).

Some indication of individual variation in predator physiology can be found in the data because the Barn Owls were kept separately. Individual 2 produced proportionately more jaws without corrosion damage than did Individual 1 (Table 2). The difference is statistically significant (binomial test, two-sided, given *p* = 0.15, *P* < 0.01).

### Feeding experiments: quantitative comparisons

We focus here on the Ferret and Barn Owl experiments, as only from these were identifiable remains recovered. The univariate cumulative link model (CLM) indicates that predator identity of *Tyto alba* rather than *Mustela putorius* has a positive (0.25) but insignificant (*P* = 0.67) influence on the extent of shaft corrosion alone (Akaike information criterion [AIC] = 120.95). Including a random effects term results in a cumulative link mixed model (CLMM) with a worse AIC (122.93), so we may discount the random effect for these data.

The univariate CLM indicates that predator identity of *Tyto alba* rather than *Mustela putorius* has a negative (–2.27) and highly significant (*P* = 0.0004) influence on the extent of crown corrosion alone (AIC = 123.71). When including a random effects term, the condition number of the matrix (Hessian) used to compute standard errors on model coefficients is > 10^6^, suggesting the model is not well defined^24^. In the absence of a way to simplify the model except by removing the random effects term, we conclude that the simpler CLM is more appropriate for crown corrosion as well.

In the multivariate CLM, the coefficients indicate that a predator identity of *Tyto alba* rather than *Mustela putorius* has a significant (*P* = 0.0013) negative influence on crown corrosion and an insignificant influence on shaft corrosion (*P* = 0.99). Predicted levels of corrosion to the crown and shaft for *M. putorius*, for this model, are (2,1), respectively. Predicted levels of corrosion to the crown and shaft for *T. alba*, for this model, are (0,0), respectively. These values represent the most probable combination of levels for crown and shaft corrosion in the two predators; for example, in *M. putorius* (2,1) is a more likely than (2,2), (1,2) or any other possible combination of corrosion levels. The marginal predictions for *M. putorius* are the same, whereas for *T. alba* they are 0 and 1, respectively. These values represent the most likely values for the level of crown and shaft corrosion considered independently; for example, in *T. alba* it is most likely that the shaft shows corrosion level 1, rather than 0 or 2, considered without regard to corrosion on the crown, and most likely that the crown shows corrosion level 0, rather than 1 or 2, considered without regard to corrosion on the shaft. Put into words, in *M. putorius* crown corrosion tends to be greater than shaft corrosion, whereas in *T. alba* the opposite is true. These results are consistent with the predictions of our hypothesis.

### Actuotaphonomy: modern owl pellets

A total of 13 jaws were recovered from six of the owl pellets. All nine jaws from four of the pellets showed no evidence of corrosion at all that is visible under the light microscope: #462 and #810 from *Tyto alba*, #712 from *Athene noctua*, and #977 from *Asio otus* (Supplementary Table S1). In contrast, one jaw each from two owl pellets—#652 and #1239, both from *Tyto alba*—showed evidence of corrosion on some teeth. Several of the front-most teeth on the dentary of *Trachylepis vittatus* (from #1239) show an abrupt constriction of the neck between the shaft and crown (Supplementary Fig. S4), i.e., ’neck’ corrosion, which on at least one tooth extended further up onto the crown (Supplementary Table S5). That corrosion should be greater on anterior than posterior teeth corresponds to the general pattern revealed by the Barn Owl feeding experiment. Several of the posterior-most teeth on the dentary of the unidentified gekkotan (from #652) also show ’neck’ corrosion (Supplementary Fig. S4). No corrosion could be discerned on the two gekkotan maxillae (same individual?) from the same pellet. The trend of greater corrosion posteriorly than anteriorly seen here is unusual. These results are not consistent with the conceptual framework.

### Applied taphonomy: Qesem Cave

**Figure 4:**
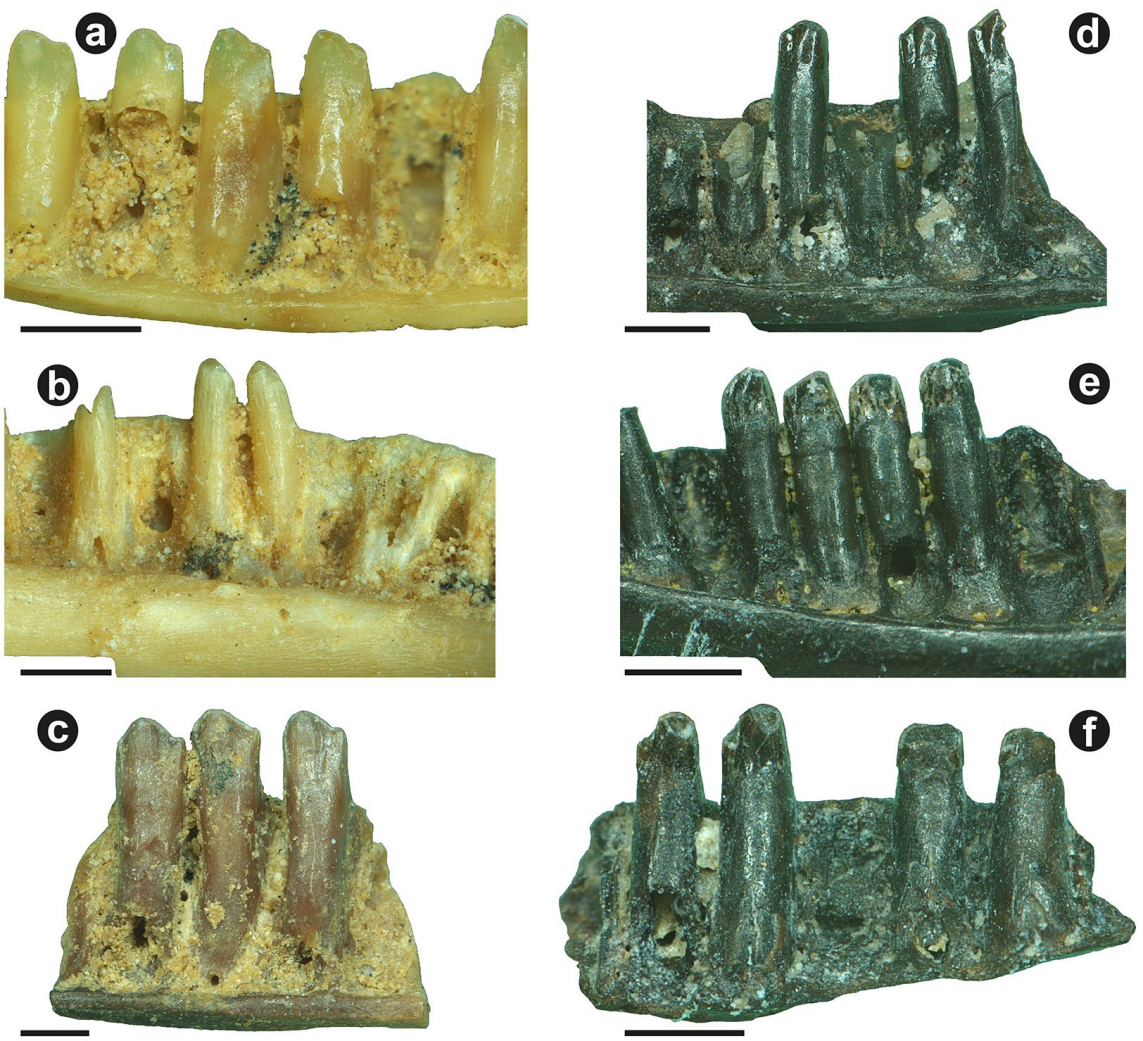
Corrosion in Quaternary lizards from Qesem Cave, Israel, and Hummal, Syria: (**a**) teeth of unidentified lacertid (Liz1-c2) from Concentration 1^62^ at Qesem Cave showing light corrosion damage; (**b**) teeth of unidentified gecko (Liz1-a5) from Concentration 1 at Qesem Cave showing no clear corrosion damage but many small, longitudinal fractures; (**c**) teeth of unidentified lacertid (Liz2-f7) from Concentration 2^62^ at Qesem Cave showing light damage attributed to abrasion, possibly trampling; (**d**) teeth of unidentified lacertid (Herp1-a8) from Hummal showing no evidence of corrosion; (**e**) teeth of unidentified lacertid (Herp1-a5) from Hummal showing no corrosion to crown (only some abrasion) but a lightly corroded shaft (type 2 corrosion); (**f**) teeth of unidentified lacertid (Herp1-b3) from Hummal showing type 2 corrosion, but where the crown has been effaced on posterior teeth, probably by spalling. Scale bars: 0.5 mm.

That corrosion is not widespread among the lower vertebrates at Qesem Cave was previously established for a part of Concentration 1^8^. This observation applies also to Concentration 2 (Table 3). Specimen Liz1-c1 is an example of such an apparently uncorroded jaw. Notably, four of the specimens, all pertaining to the legless lizard *Pseudopus*, could not adequately be assessed because the degree of pleurodonty is too low for cheek teeth (i.e., there is virtually no tooth shaft) and because abrasion, presumably due to diet, was too great on the broad, flat tooth crowns. However, one specimen of a lacertid from Concentration 1 (Liz1-c2) shows very light corrosion to the shaft as well as crown (Fig. 4a). A gekkotan jaw from the same concentration shows no clear evidence of corrosion to the teeth, but the shafts are permeated by many very fine-scale fractures that generally run parallel to the tooth’s long axis (Fig. 4b); all three remaining tooth bases are characterized by a midline split that ascends the lingual surface of the shaft halfway from the base, similar to splits observed in the jaws coming from the Ferret.

**Table 3:** Summary of corrosion damage to teeth of pleurodont lizards from Qesem Cave, Israel, and Hummal, Syria.

Concentration	*N*	*n* showing corrosion (C) to crown	*n* showing corrosion (C) to shaft	C(crown) > C(shaft)	C(shaft) > C(crown)
Qesem Conc. 1	3	1 or 2	1 or 2	0	0
Qesem Conc. 2	11	0	0	NA	NA
Hummal	33	10	13	5	6

Concentration numbers for Qesem Cave follow Smith et al.^64^. Four specimens of *Pseudopus* sp. from Concentration 2 were excluded (see text).

The specimens from Concentration 2 must be examined with special care because this concentration is located toward the center of the cave where compaction and trampling are a potential issue. For instance, lacertid jaw fragment Liz2-f7 shows light damage over the entire lingual surface of the teeth (Fig. 4c); however, the surfaces bordering the interdental spaces, which would not have been exposed to abrasion by sediment, are unaffected. This specimen is suggestive of trampling damage, rather than corrosion. No specimen from Concentration 2 showed clear evidence of corrosion.

The modal extent of corrosion to crown and shaft is (0,0), respectively. This result is the same as the predicted values of the multivariate cumulative link model. Consequently, the virtual lack of corrosion shown by the Qesem specimens is consistent our experimental feeding with *Tyto alba*. However, raptors other than *T. alba* were not examined, so other potential strigiform predators cannot be excluded. The heavy crown corrosion that characterized jaws consumed by *Mustela putorius* in all statistical models is absent, suggesting that any predator of similar of physiology is not responsible for the accumulations. To the extent that other Carnivora share the same physiology, this would further support the contention^8^ that it was not a mammalian carnivore.

### Applied taphonomy: Hummal

Of the pleurodont lizard jaws from Hummal, 20 of 33 (61%) showed no clear evidence of corrosion (Fig. 4d). The remainder (13 of 33, or 39%) did. Corrosion is more common on the shaft than the crown (Table 3). It is somewhat more common that damage to the shaft exceeds damage to the crown (6 cases) than the converse (5 cases). Among the 5 cases in which damage to the crown exceeds that to the shaft, only 1 of them approaches type 1 corrosion (Fig. 1). In contrast, among the 6 cases in which damage to the shaft exceeds that to the crown, 5 of them closely correspond to type 2 corrosion (Fig. 4e). Furthermore, where crown damage exceeds shaft damage, this is frequently attributable to complete loss of the enamel; loss of the enamel may in many of these cases have occurred not by direct corrosion of the enamel but rather by the spalling of enamel flakes where these are undercut by corrosion to the dentine. Intermediate cases—where enamel is present on some teeth but (partly) spalled off on other teeth—include specimen Herp2-b3 (Fig. 4f).

The modal extent of corrosion to crown and shaft is (0,1), respectively. Heavy crown corrosion characterizes lizard jaws consumed by *Mustela putorius* in all statistical analyses, so this result stands in strong contrast to that. A predator of similar end-member physiology can safely be excluded. Furthermore, in our experiments with artificial gastric fluids, exposure to high-acid, high-enzyme conditions proved to be extremely destructive of lizard teeth (Supplementary Fig. S2w), and a predator with a similar digestive physiology, as seen in extant diurnal raptors (Supplementary Note), can also be excluded. The Hummal results are also not identical to the joint crown-shaft prediction for *Tyto alba* (0,0). In sum, the results are consistent with a predator with similar low-acid, high-enzyme physiology but slightly greater acid content, such as a more destructive owl, where gastric pH may range from 2.2 to 2.5 (Supplementary Note)^3,25^.

### Applied taphonomy: Paleogene sites

**Figure 5:**
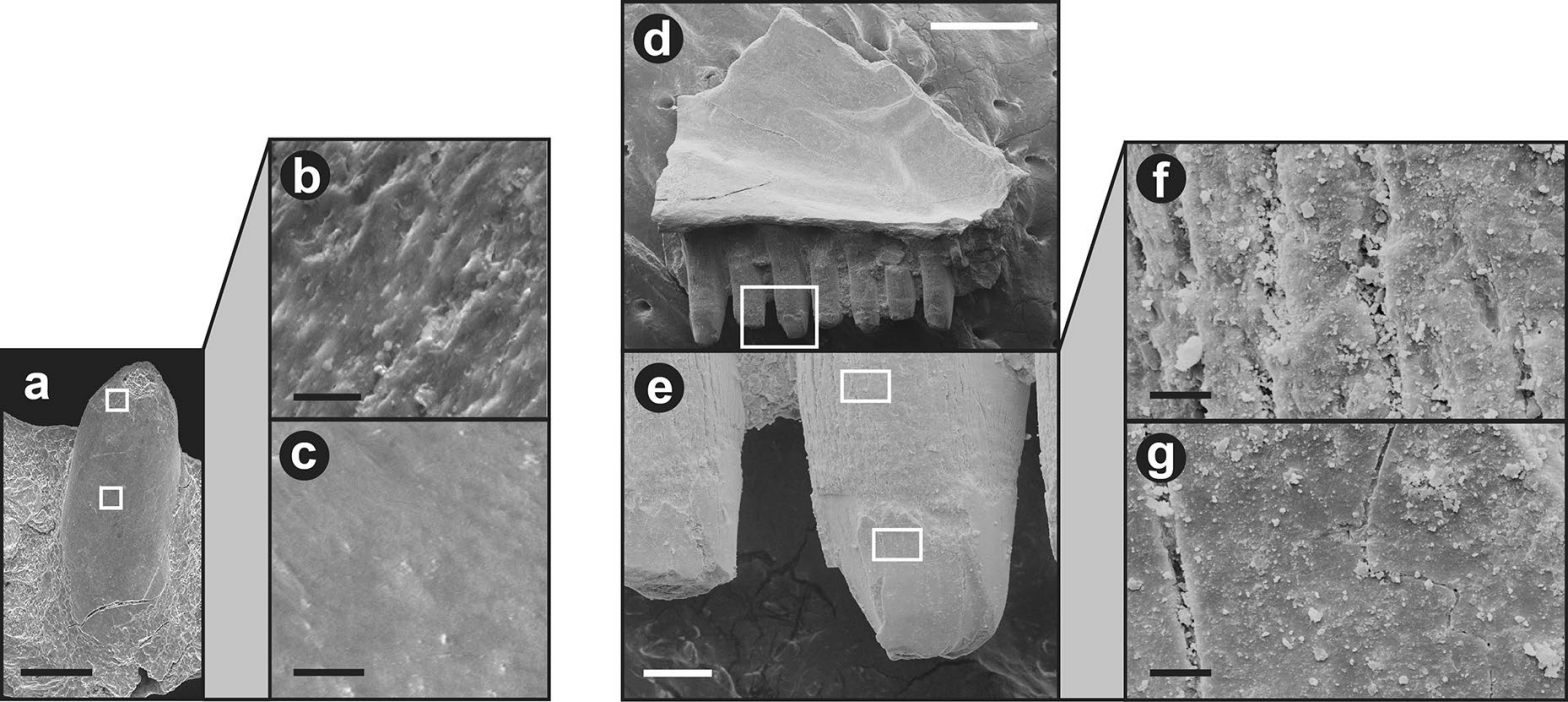
Corrosion damage to teeth of pleurodont lizards from the early Paleogene of Wyoming, USA: (**a**–**c**) type 1 corrosion (from Big Multi), in which the crown has been completely destroyed (note thinning toward the tip) but the shaft is undamaged, and (**d**–**f**) type 2 corrosion (from Turtle Graveyard), in which the crown enamel is undamaged (except by breakage, perhaps during screenwashing) but the shaft is corroded. Note also the midline fracture at the lingual base in the latter.

The number of specimens in each of the seven localities showing evidence of corrosion is given in Table 4. Corrosion was observed in a high proportion of lizard jaws in all seven localities, with frequencies between 66 and 86%. The locality with the highest frequency was Level S, with 85.7%. There is no outlier on the lower end. Both type 1 (Fig. 5a–c) and type 2 (Fig. 5d–f) corrosion were commonly observed. ‘Neck’ corrosion (see above) was also observed but was much rarer. The proportion of specimens falling in damage categories varies strikingly by locality (Table 4). The ratio of type 1 to type 2 corrosion varies from about 1:1 to 1:3; thus, while type 2 corrosion is more common, type 1 is a significant minority (25–50%). Only at Big Multi did specimens showing type 1 corrosion outnumber those showing type 2. At Dorsey Creek Quarry the bone concentration is spatially restricted^26^, and nearly all lizard jaws (92%) show type 2 corrosion.

**Table 4:** Summary of corrosion damage to lizard teeth from seven Paleogene localities in Wyoming, USA.

Paleogene fossil locality	*N*	C(crown) > C(shaft)	C(shaft) > C(crown)	‘Neck’ corrosion
Big Multi	55	20	16	0
Castle Gardens	145	29	67	1
Dorsey Creek	91	6	66	0
Level M	154	28	78	9
Level O	146	46	51	7
Level S	42	7	27	2
Turtle Graveyard	207	48	79	10

These numbers generally cannot be directly compared with the foregoing, because they pertain only to identified specimens. Thus, if corrosion was present but so extensive that it precluded identification, then it would not be counted.

Intrajaw variation in corrosion is noteworthy. Most cases involve intrajaw variation in the extent of corrosion, as was also noted in the experiments. There are also two cases in which more than one corrosion type simultaneously occur in a single jaw. In specimen UCMP (University of California Museum of Paleontology) 167586 (from Castle Gardens), an anterior tooth shows type 1 corrosion while posterior teeth show type 2; a similar but less marked case is presented by UCMP 215567 (from Level M). The laboratory experiments with artificial gastric fluids as well as the Quaternary site of Hummal provided previous examples of this.

## Discussion

Here we address the following questions: (1) Is the proposed framework for digestive corrosion on lizard teeth, and more specifically the corrosion sequences, borne out by the experiments, and if so, what implications does this have for experimental digestion in taphonomy? (2) How can the results be applied to actual Quaternary assemblages, with the goal of identifying predators in a taxonomic sense, and what additional studies could further this aim? (3) How can the results be applied to older assemblages, where the likely accumulator is extinct? (4) What can we infer about the evolution of digestive strategies on the basis of our literature review and experiments?

With regard to the first question, the results are broadly consistent with our hypotheses. In the laboratory experiments, cases of type 2 corrosion were concentrated in the low-acid, high-enzyme portion of parameter space, as predicted, although quantitative support was mixed. The feeding experiments with Ferrets (high-acid, presumed low-enzyme) and Barn Owls (low-acid, presumed high-enzyme) predators provided further support, as Ferrets tended to corrode the crown more heavily, and Barn Owls the shaft. Thus, we find support for the framework we established in which both the nature of the corrosive agent and the type and distribution of prey tissues influence corrosion patterns.

Patterns resulting from our experiments included idealized type 1 and type 2 corrosion (Fig. 1), as predicted, but there was considerable variation. The idealized types were not so common in the laboratory or feeding experiments, for which reason we focused, for quantitative analyses, on whether crown corrosion exceeded shaft corrosion or vice versa. Cases in which two corrosion patterns are simultaneously shown by the same jaw were extremely rare, and they could be explained as the result of a trend where the lizard jaws were sufficiently complete to study intrajaw variation. Some cases in which shaft damage exceeded crown damage were found among high-acid, low-enzyme systems (Ferret), and the converse as well. The variability of the results confirms that statistical analysis is a crucial part of the study of corrosion patterns, like other taphonomic phenomena, even or especially in non-experimental contexts^25^.

This variability may arise from a number of sources. First, the composition of prey animals may be variable because of ontogenetic stage or state of health. Second, idiosyncratic factors such as where the prey tongue is located (e.g., if it sticks out of the mouth and protects some tooth crowns) and interaction with other food stuffs may play a role. Third, predator behavior may play a decisive role. Our laboratory experiment showed that whether the lizard mouth is open when exposed to the corrosive fluids has a dramatic influence on the resulting corrosion (Supplementary Methods). Mammalian predators may comminute their prey much more than owls (as shown here by the Ferret and especially Honey Badger), which could change the proportion of jaws that are directly exposed to corrosive fluids. Additionally, some predators decapitate the prey and may simply let the heads fall to the ground, where they might commingle with bones derived from pellets^3^. Fourth, predator physiology (acidity, enzyme activity, gastric residence time) is not constant but changes in response to overall health, feeding state and age. Finally, inter-individual variation may also play a role. In the Barn Owl experiment, we observed statistically significant differences between the two individuals in the proportion of uncorroded specimens they produced. That the Barn Owl that more readily ate the anoles, and was more destructive, was an old (17 yr) blind (due to old age) male, whereas the other was a sighted, 11-year-old female, is possibly related, but we cannot be certain.

In addition to the predicted corrosion patterns, ’neck’ corrosion was observed on lizard teeth derived from Barn Owl pellets as well as in the scat of Ferrets, so it can presumably be induced by predators that normally show high-acid, low-enzyme as well as low-acid, high-enzyme conditions. In the former case, ’neck’ corrosion may be an intermediate stage on the way to type 1 corrosion. In the latter case, ’neck’ corrosion may be a more advanced stage beyond type 2 corrosion, where digestion of the gingiva has not proceeded strongly. Therefore, we see no reason to associate ’neck’ corrosion exclusively with one or the other physiological end-members.

The study of wild-collected owl pellets, in contrast to the experiments, did not support our hypotheses. Corrosion was detected only 2 of the 13 lizard jaws recovered from owl pellets, both regurgitated by *Tyto alba*, and the detected ‘neck’ corrosion (sometimes extending onto the lower crown) had not been predicted. The sample size (2) is too low to draw any conclusions from these observations. The jaws of the gekkotan in one pellet were so small that they were somewhat translucent, and it was also not possible to clean them fully, so more subtle evidence of corrosion, especially to the non-vitreous (hence, less shiny) shaft, might go unnoticed. Additionally, the prey species were different in the two pellets, which adds a further, uncontrolled element to these natural experiments. It would be desirable to study a large, preferably monospecific sample of lizard jaws from pellets (and scat) from wild predators, especially owls, to further test our conceptual framework under more natural conditions. Such assemblages have occasionally been reported in the literature^27^.

To date, all enzyme-acid experiments to study corrosion were conducted on isolated, fresh bones^16,23,28^, and those experiments using whole or partial carcasses were focused on digestion in general, not corrosion^29,30^. If the soft-tissue environment of skeletal tissues as well as their composition influence corrosion patterns, then new experiments on microvertebrate prey may be illuminating. Much phenomenological work on the remains of small mammals ingested by predators, for instance, has considered whether corrosion is visible on postcranial skeletal elements^3^. The ends of the bones, even if they are fully formed, may be covered with a cartilage cap, and the bones as a whole are normally covered by a thick layer of fur, skin, muscle and other soft tissues. Exposure of bones to gastric fluids could occur in two ways: digestion of the flesh, or dismemberment of the body. The first of these is analogous to the digestion of the gingiva explored in the present work. The second of these has no parallel in the above work on teeth. Severing of any body part will break the bones that supported it and immediately expose the broken ends to corrosive fluids in the stomach, regardless of whether the flesh is digested^3^. Other pathways (such as the penetration of gastric fluids to long-bone articular ends via the marrow cavity or between muscle fascicles, or digestion of long-bone articular surfaces by enzymes as well as acid) may be important. Additionally, other aspects of bone modification (flaking, curling, thinning) have been observed to differ among predator species^3,31^. The extent to which these aspects can be explained by combinations of acid, enzymatic activity, and breakage is unknown.

With regard to the second question—that of application in the Quaternary—present data suggest that the three major groups of microvertebrate bone accumulators normally considered in Quaternary taphonomy—mammalian carnivores, owls, and diurnal raptors—are physiologically distinct (Supplementary Note). Mammalian carnivores belong to the high-acid, low-enzyme physiological end-member, and the results of our studies suggest that they produce more extensive damage to the tooth crowns of pleurodont lizards than to the shafts. Owls belong to the low-acid, high-enzyme physiological end-member, and the results of our studies suggest that they produce more extensive damage to the tooth shafts of pleurodont lizards than to the crowns. Here, we may further distinguish Barn Owls from other owls, for Barn Owls have distinctly lower gastric acidity and therefore tend to cause even less damage overall. Indeed, based on our feeding experiments the joint prediction of the level of corrosion by Barn Owls to crown and shaft was (0,0). Finally, diurnal raptors have high-acid, high-enzyme conditions of the gastric fluids, and the results of our laboratory studies suggest that they are highly destructive of pleurodont lizard teeth. These conditions are not likely to produce identifiable lizard dental specimens. Similarly, pellet analyses have generally shown that most diurnal raptors are highly destructive of bone^2,3,25,30,32^.

A more detailed application to particular assemblages will require further studies. One goal would be to elucidate how physiological properties (particularly gastric pH and enzyme activity) vary within and amongst different taxa. Additionally, further feeding experiments with a variety of captive predators in which prey species identity is constant would enable detailed statistical and observational comparisons between different predators. Finally, studies of large samples of lizard jaws from wild-collected pellets and scat produced by known predator species will be required to fully understand the kinds of damage to lizard teeth (or other body parts) and the range of skeletal variation and its causes.

In addition to the corrosion types discussed above, the overall degree of damage should be considered. Mammalian predators very much larger than their prey cannot be considered as the origin of bone accumulations, because they chew their prey so thoroughly that little or nothing remains (as in the Honey Badger here). Our study corroborates the previous works by Andrews, Fernández-Jalvo and others^3,25,33,34^ that have shown that Barn Owls corrode their prey very lightly.

With regard to the third question—that of application outside the Quaternary—we emphasize that our approach focused on physiological, rather than taxonomic, classes and in that sense is taxon-free. In theory, physiological properties can be inferred for extinct taxa using phylogenetic comparative methods and an appropriate phylogenetic framework, and our approach could therefore also be used to assess candidate predators in Deep Time. However, the digestive physiology of most extant predators is inadequately known, and more data must be acquired on extant taxa to infer anything beyond the three broad groups of predators noted above.

Even if particular predators cannot yet be identified for our Paleogene sites, our observations provide insight into the origin of our microvertebrate accumulations. The high proportion of corrosion observed on lizard jaws from all Paleogene assemblages is consistent with a predatorial origin of the accumulations from which they derive. Fernández-Jalvo and Andrews^35^ and Fernández-Jalvo et al.^19^ noted that alkaline corrosion, as might occur in some soils, will mostly affect bone and dentine. This is due to its caustic effects on organic matter, such as collagen, which is effectively absent in enamel. Indeed, enamel thins quickly toward the shaft of lizard teeth, and apparent corrosion to the shaft of lizard teeth caused by exposure to bleach corresponds exactly to the prediction of type 2 corrosion^11^ (Fig. 1). But alkali corrosion cannot explain type 1 corrosion, in which the enamelled tooth tips are preferentially destroyed, nor ’neck’ corrosion, which is highly spatially restricted, nor can it explain data from our Paleogene fossil localities, in which several corrosion patterns co-occur.

While the common occurrence of corrosion on specimens studied here is consistent with the notion that one or more predators contributed to the concentration of microvertebrate bones there, it does not preclude a hydrologic influence. That is, a concentration of bones accumulated by a predator may subsequently be reworked and deposited away from the original site of accumulation. Pellets are only very rarely preserved in the fossil record^36^.

The balanced co-occurrence of different corrosion types in most of the Paleogene samples suggests that there may have been more than one agent of accumulation. Dorsey Creek Quarry was an outlier in that nearly 90% of all lizard jaws showed type 2 corrosion (Fig. 1). In light of our experiments, this result suggests that a low-acid, high-enzyme predator such as an owl was responsible for the accumulation. Owls are known in North America since the Paleocene^37^. In contrast, Silcox and Rose^26^ made the preliminary suggestion that carnivoran mammals were responsible for the collection. Insofar as known carnivorans fall into the high-acid, low-enzyme end-member physiology, our results are inconsistent with that suggestion. If a single strigiform predator was responsible for the accumulation, then the unusual taxonomic composition—dominated by lipotyphlan insectivores^26^—could provide insight into predator paleobiology.

With regard to the last question—the evolution of digestive strategies—our experiment with artificial gastric fluids highlighted a critical limitation that itself may provide insight into the evolution of digestive strategies. In contrast to Christiansen et al.^29^, who produced extensive degradation of fish carcasses after only 10 h in comparatively low-pepsin solutions, our experiments with unrealistically high-pepsin solutions did not mimic that degradation or routinely reproduce type 2 corrosion (Fig. 1), as predicted. While an explanation for this discrepancy is not immediately clear (differences between fish and lizard skin and other tissues could play a role), a further explanation may be entertained, one rooted in a basic difference between bird digestion and digestion in other vertebrates.

Generally speaking, there are two possible ways of dealing with indigestible dietary components (Supplementary Fig. S5). They may be passed into the intestine after a stint in the stomach and excreted with feces, or they may be regurgitated^38^. As the former strategy is present in all major extant clades of jawed vertebrates (Gnathostomata) except for birds (Supplementary Note), it appears to be the plesiomorphic (evolutionarily primitive) strategy. Here, defleshing of a carcass is a spatially protracted process, and most of the enzymatic breakdown occurs in the small intestine. This strategy may be referred to as *enteric digestion* (Supplementary Fig. S5).

The digestive system in birds is profoundly transformed in association with the evolution of active flight^39–41^. In some taxa, prey animals may be stored briefly in a distensible esophagus (crop), but usually carcasses quickly enter the gizzard (ventriculus, muscular stomach), where defleshing takes place^42,43^. The pyloric opening to the small intestine is small and cranially placed in birds of prey, limiting the size of particles that can enter the intestine^39,42,44,45^. Therefore, indigestible contents of the diet, particularly keratin and bones, do not enter the intestine but rather are formed into a pellet and ejected through the mouth^32,46–48^, a uniquely avian feature^43^. The propensity to form pellets apparently evolved within theropod dinosaurs (Supplementary Note) and is found in numerous unrelated clades of extant birds^38,49^. Pellet egestion in birds may be closely related to emesis in lower vertebrates^50^, as may occur sporadically. However it evolved, pellet egestion means that a carcass must be defleshed early in the digestive process. If any nutrient is to reach the intestine to be absorbed, it must be released in the stomach. This strategy, in which a more significant proportion of the chemical breakdown occurs in the stomach, may be referred to as *gastric digestion* (Supplementary Fig. S5).

In enteric digesters, predigestion in the stomach is not as important. Consistent with this, proteolytic activity in the stomach of mammalian carnivores, although it may vary in response to several factors^51^, has been found to be only a third that of birds of prey at comparable stages of digestion^23,45,52^. In contrast, in gastric digesters defleshing must take place in the stomach, and it is not surprising that proteolytic activity in the stomach is high in birds of prey^45^. Moreover, gastric digestion in birds is accompanied by a unique physiological mechanism, intestinal reflux, whereby antiperistaltic waves^53^ empty nearly the entire contents of the upper intestine into the muscular stomach^40^. With these contents, a bevy of downstream enzymes from the pancreas and liver enters the stomach and, with rising pH^42^, becomes active^40,53,54^. These additional enzymes probably contribute to a more complete chemical breakdown in the stomach than would otherwise be possible. Intestinal reflux likely evolved at the latest by crown Aves^40^ (Supplementary Note).

Our simple experimental model with artificial gastric fluids included only pepsin, the dominant gastric enzyme in vertebrates. The failure of the low-acid, high-enzyme solutions to achieve significant lizard carcass degradation or reliably produce type 2 corrosion (Fig. 1) suggests that our experimental model did not capture critical aspects of digestion in birds. The physiological review above suggests a crucial role for downstream enzymes, entering the stomach by retroperistalsis, in digestion in birds of prey, and by extension, other birds. A more realistic, two-step experimental set-up that incorporates these enzymes is called for.

## Conclusions

The present paper builds on the tradition of controlled experimentation in taphonomy^55^. It furthermore highlights a unique role that lower vertebrates may play in the elucidation of fossil assemblages. Taphonomic phenomena in reptilian remains are poorly studied, so much so that Lyman^56^ lamented being able to devote but a single page to them. On the one hand, this is hardly tragic if lower vertebrates possess no properties that distinguish them as taphonomic objects, collectively or individually, from mammals. Yet this does not appear to be the case^57^.

The conceptual framework of corrosion developed here exploits the pleurodont tooth implantation common in lizards. We deduced hypotheses of corrosion patterns and then tested our hypotheses using experiments with artificial gastric fluids and real predators. Statistical analysis, particularly of the relative degree of corrosion to the tooth crown and shaft, was helpful in the face of variability in the results. Our results support the notion that the nature of corrosive agents and the composition of the tissues on which they act both influence corrosion patterns on lizard jaws.

At present, three broad categories of predators can currently be distinguished on the basis of gastric physiology: mammalian carnivores (high-acid, low-enzyme), owls (low-acid, high-enzyme), and diurnal raptors (high-acid, high-enzyme). These categories, while coarse, provided some insight into the origin of the microvertebrate accumulations examined here. Further studies on extant predators are needed in order to refine our approach, particularly from controlled feeding experiments and from wild-collected pellets and scat. These data would facilitate a finer taxonomic understanding. Furthermore, more data on the gastric physiology of extant predators would help to predict the kinds of corrosion patterns to be expected from extinct predator species, in a phylogenetic context.

The mammalian digestive strategy, in which a significant fraction of prey breakdown takes place in the intestine (enteric digestion), appears to be primitive for jawed vertebrates. This became transformed in the evolution of birds, and in extant raptors a more significant fraction of prey break down takes place in the stomach (gastric digestion). In addition to changes in stomach chemistry, this transformation is accompanied by unique physiological mechanisms such as pellet production and intestinal retroperistalsis (bringing downstream enzymes into the stomach), which might play an important role in prey digestion.

## Materials and methods

Lizards used in experiments were all *Anolis carolinensis* (Green Anole) of similar body size. Experiments were conducted in accordance with the Tierschutz-Versuchstierverordnung (Germany) or the Wildlife Protection Act of 1955 (Israel Nature and National Park Authority permit no. 40162/2013). In general, corrosion was recognized as a roughening of the normally smooth tooth surface that affects not only exposed surfaces but also protected surfaces like those between the teeth. This is visible when incident light reflects off the tooth surface and is confirmed by scanning electron microscopy (SEM). Abrasion was distinguished from corrosion in that it affects only salient or projecting parts of teeth and not, for instance, the surfaces bounding the interdental space^2,18^. Fisher^17,18^ found that the enamel could be preserved interdentally where adjacent, corroded teeth were closely apposed. The teeth do not touch in *A. carolinensis*.

### Artificial gastric fluids

Our conceptual framework has two active components, HCl and enzymes. For experimental purposes, the enzyme component was represented by pepsin, the main digestive enzyme in the stomach. Fresh carcasses of anoles (not isolated bones) were exposed to a variety of simulated gastric fluids consisting of HCl and pepsin mixed with distilled water (Supplementary Methods). Duration of exposure, pH and pepsin concentration were varied in ways designed to mimic natural predators (mammalian carnivores, owls, and diurnal raptors). Qualitatively, we assessed whether the corrosion patterns corresponded to the predicted patterns, type 1 or type 2, and whether any other, unpredicted patterns emerged. Quantitatively, we counted in how many specimens corrosion was greater to the crown of the teeth than the shaft, and vice versa; this measure is more generally applicable, as it covers cases that do not cleanly fit into the predicted patterns. Note that by definition, in any specimen showing type 1 corrosion, crown corrosion is greater than shaft corrosion, and in any specimen showing type 2 corrosion, shaft corrosion is greater than crown corrosion.

### Feeding experiments

We selected four predator species: Ferret (*Mustela putorius*), Honey Badger (*Mellivora capensis*), Kestrel (*Falco tinnunculus*) and Barn Owl (*Tyto alba*). Available data suggest that the Ferret represents the high-acid, low-enzyme physiological end-member, and that the Barn Owl represents the low-acid, high-enzyme end-member (Supplementary Note). Furthermore, available data suggest that the Kestrel has high-acid, high-enzyme gastric fluids. The physiology of the Honey Badger stomach is unknown.

The Ferret experiment was conducted at the Paul Ehrlich Institute for Vaccines and Biomedicines in Langen, Germany. Thirty-five anoles were euthanized and decapitated. The heads and bodies of 33 were fed to 4 freshly captive ferrets over the course of 4 days. We also offered 2 of the lizard heads to an individual that had long been held at the institute, but it refused the novel food item. Due to concerns about meal size (ultimately unfounded), we began with the heads and fed the bodies later. The 4 individuals were held in only 2 separate cages (2 per cage), so that an individual attribution of particular lizard remains is not possible. Feeding behavior was observed. All scat was collected and macerated in water for up to two weeks to separate the bones from the other fecal material. The bones were then picked out under a binocular microscope.

The other feeding experiments were conducted at the I. Meier Segals Garden for Zoological Research at Tel Aviv University, Israel. Seventy-nine anoles were euthanized with CO_2_ and fed to two captive predator species: Honey Badger (1 individual, which otherwise was fed chicks) and Barn Owls (2 individuals in separate cages, which otherwise were fed mice). The Honey Badger was fed 11 anoles in addition to its staple food of chicks (*Gallus gallus domesticus*), as the anoles did not suffice to feed it. Five Kestrels were fed 14 anoles. One Barn Owl was fed 11 anoles and the other 4 anoles. Feeding behavior was observed. Pellets and scat were collected and stored in 70% EtOH solution for several months, during which they were teased apart and anole skeletal elements were picked out. No maxillae or dentaries were recovered from the Kestrel pellets.

Jaw fragments were divided into quadrants (right and left dentaries, right and left maxillae) and examined under a light microscope for evidence of corrosion. We counted specifically teeth in the middle of the jaw (positions 10–15 of about 25). Select specimens were also studied under SEM (see above). We collected the same type of data as in the experiments with artificial gastric fluids (see above). Furthermore, we used a simplified ordinal scale of 0–1–2^25^ to describe the extent of corrosion to the crown and the shaft, where the values were described as follows: 0—no corrosion visible, 1—corrosion visible but less extensive than next level, 2—corrosion extensive (enamel of crown destroyed or decalcified, or shaft considerably thinned with enlarged nutrient foramen if basally extensive). The crown and shaft were scored separately for each individual specimen (‘Subject’ or ‘Alt_subject’) taken from each predator species (‘Species’) and individual (‘Individual’). These data can be found here: https://doi.org/10.5061/dryad.x69p8czhn.

### Statistical analyses

We used univariate and multivariate cumulative link models (CLMs), as implemented in the packages “ordinal”^58^ and “mvord”^59^ in R to study the ordinal data. The element with the highest *N* for each predator species (right dentary for *Mustela putorius*, left dentary for *Tyto alba*) was selected. Comay and Dayan^25^ used univariate CLMs to study corrosion and breakage phenomena in raptor pellets, including differences among observers. We did not study the last effect, but it should not influence the results because observations from only a single observer (KTS) are included. The package ordinal allows for random effects (here, predator individual or cage), thus cumulative link *mixed* models (CLMMs). Selection among hierarchical models (in the present case, univariate CLMs of corrosion with and without random effects) was accomplished using the Akaike information criterion (AIC), where lower values of AIC indicate a better fit. The general formula for the linear model contained one ordinal response or dependent variable (Corrosion, to crown or shaft) and one categorical predictor or independent variable (Species), with or without a random effect (Individual): *Corrosion* ∼ *Species (*+ *Individual)*.

The simultaneous study of corrosion to crown and shaft, with the potential for covariation, necessitated the use of a multivariate model, in which case there were two simultaneous response variables: *Corrosion_Crown* + *Corrosion_Shaft* ∼ *Species*. Random effects have not yet been implemented in the mvord package, but since our initial analyses using CLMMs did not support an important random effect, this limitation should not influence the results. The *predict* functions in mvord were used to predict joint and marginal probabilities and classes for the response variables given species identity (the independent variable). These predictions were compared to the corrosion modes discovered in the Quaternary fossil sites.

The code and output of analyses can be found here: https://doi.org/10.5061/dryad.x69p8czhn.

### Actuotaphonomy: modern owl pellets

Rarely lizards can make up a large percentage of the diet in owls^8^. As part of a taphonomic study of extant owl pellets in Israel, Comay^60^ studied 3001 pellets and prey assemblages from five owl species in Israel (Table 2). Six of these pellets contained at least one jaw of a pleurodont lizard (Supplementary Table S2).

### Applied taphonomy

Pleurodont lizard jaws identified from two different stratigraphic intervals were examined: (1) the Pleistocene of Qesem Cave, Israel, and Hummal, Syria; and (2) the early Paleogene of Wyoming, USA (Supplementary Table S3).

Qesem Cave is an Acheulo-Yabrudian site in the Judean Hills of Israel dated to 420–200 kya^61^. Its archaeological findings document several technological innovations reflecting modern human behavior, such as systematic tool production, selective flint quarrying and recycling, and an association between materials and tool types. Microvertebrate concentrations 1 and 2 at Qesem Cave are exceedingly rich^62^. Based on study of the lower vertebrates, Smith et al.^8^ proposed a Barn Owl as the accumulator, but based on study of micromammals, Comay and Dayan^63^ suggested a more destructive owl. Most of the lizards from both concentrations in this locality represent the acrodont species *Chamaeleo chamaeleon* and *Stellagama stellio*, in which the teeth are fused with the apex of the jaw parapet. The assumption of the conceptual model—that there is a tooth shaft only covered lingually by the gingiva—is therefore not fulfilled in these species. Therefore, these acrodont specimens were excluded from further consideration. The 18 pleurodont lizard jaws could be examined. These specimens were recovered by screenwashing under water and were picked individually from concentrate.

The site of Hummal is situated in the El Kowm region, in the arid interior of Syria. Numerous artesian springs formed the basis of a more or less continuous occupation of the region during the Pleistocene^64^. The deposits of Hummal represent conditions ranging from marshy to lacustrine, as a consequence of changing water table and rapid sedimentation rates, creating finely stratified layers with well-preserved archaeological remains. The microvertebrates from Hummal in this study originate from Unit G / Layer 17. Acrodont lizards occur here also, but for reasons noted above, these were excluded from the present study, which treats only the 33 pleurodont lizard jaws. These specimens were recovered by screenwashing under water and were picked individually from concentrate.

We also chose seven early Paleogene localities from Wyoming, USA, in which to study corrosion. These are five localities in the Washakie Basin—Big Multi Quarry and Levels M, O, and S and Turtle Graveyard—and two localities in the Bighorn Basin, Castle Gardens and Dorsey Creek Quarry. The localities represent a variety of different lithologies. We studied a total of 840 pleurodont lizard jaws from these localities. The specimens were recovered by screenwashing under water and were picked individually from concentrate. Since counts here are based on *identified* specimens, and extensive corrosion might preclude identification in some cases, the numbers reported must been seen as minima.

These data can be found here: https://doi.org/10.5061/dryad.x69p8czhn.

## Supplementary Information

Supplementary Information 1.

## References

[1] Mellett JS (1974). Scatological origin of microvertebrate fossil accumulations. Science.

[2] Mayhew DF (1977). Avian predators as accumulators of fossil mammal material. Boreas.

[3] Andrews P (1990). Owls, Caves and Fossils.

[4] Fernández-Jalvo Y, Andrews P (2016). Atlas of Taphonomic Identifications; 1000+ Images of Fossil and Recent Mammal Bone Modification.

[5] Lev M, Weinstein-Evron M, Yeshurun R (2020). Squamate bone taphonomy: A new experimental framework and its application to the Natufian zooarchaeological record. Sci. Rep..

[6] Evans SE (2003). At the feet of the dinosaurs: The early history and radiation of lizards. Biol. Rev..

[7] Bailon S, Rage J-C (1992). Amphibiens et reptiles du Quaternaire. Relations avec l'homme. Mém. Soc. géol. France, n. s..

[8] Smith KT, Maul LC, Barkai R, Gopher A (2013). To catch a chameleon, or actualism vs. natural history in the taphonomy of the microvertebrate fraction at Qesem Cave, Israel. J. Arch. Sci..

[9] Terry RC (2004). Owl pellet taphonomy: A preliminary study of the post-regurgitation taphonomic history of pellets in a temperate forest. Palaios.

[10] Zaher H, Rieppel O (1999). Tooth implantation and replacement in squamates, with special reference to mosasaur lizards and snakes. Novitates.

[11] Delgado S, Davit-Béal T, Allizard F, Sire J-Y (2005). Tooth development in a scincid lizard, *Chalcides viridanus* (Squamata), with particular attention to enamel formation. Cell Tissue Res..

[12] Poole DFG (1957). The formation and properties of the organic matrix of reptilian tooth enamel. Quart. J. Microsc. Sci..

[13] Barrington EJW (1942). Gastric digestion in the lower vertebrates. Biol. Rev..

[14] Cash RA (1974). Response of man to infection with *Vibrio cholera.* I. Clinical, serologic, and bacteriologic responses to a known inoculum. J. Infect. Dis..

[15] Withers PC (1992). Comparative Animal Physiology.

[16] Fernández-Jalvo Y, Andrews P, Sevilla P, Requejo V (2014). Digestion versus abrasion features in rodent bones. Lethaia.

[17] Fisher DC (1981). Crocodilian scatology, microvertebrate concentrations, and enamel-less teeth. Paleobiology.

[18] Fisher DC (1981). Taphonomic interpretation of enamel-less teeth in the Shotgun local fauna (Paleocene, Wyoming). Contrib. Mus. Paleont., Univ. Mich..

[19] Fernández-Jalvo Y, Sánchez-Chillón B, Andrews P, Fernández-López S, Martínez LA (2002). Morphological taphonomic transformations of fossil bones in continental environments, and repercussions on their chemical composition. Archaeometry.

[20] Berna F, Matthews A, Weiner S (2004). Solubilities of bone mineral from archaeological sites: The recrystallization window. J. Arch. Sci..

[21] Courts A (1960). Structural changes in collagen: The action of alkalis and acids in the conversion of collagen into eucollagen. Biochem. J..

[22] Harkness MLR, Harkness RD, Venn MF (1978). Digestion of native collagen in the gut. Gut.

[23] Cummings JH, Duke GE, Jegers AA (1976). Corrosion of bone by solutions simulating raptor gastric juice. Raptor Res..

[24] Christensen, R. H. B. A tutorial on fitting cumulative link mixed models with clmm2 from the ordinal package. https://cran.r-project.org/web/packages/ordinal/vignettes/clmm2_tutorial.pdf (2015).

[25] Comay O, Dayan T (2018). Taphonomic signatures of owls: New insights into micromammal assemblages. Palaeogeogr., Palaeoclimatol. Y, Palaeoecol..

[26] Silcox, M. T. & Rose, K. D. in *Eocene Biodiversity: Unusual Occurrences and Rarely Sampled Habitats* (ed Gunnell, G. F.) 131–164 (Kluwer Academic/Plenum Publishers, 2001).

[27] Brain CK (1981). The Hunters or the Hunted? An Introduction to African Cave Taphonomy.

[28] Denys C, Fernández-Jalvo Y, Dauphin Y (1995). Experimental taphonomy: Preliminary results of the digestion of micromammal bones in the laboratory. C. R. Acad. Sci. Paris IIA.

[29] Christiansen JS, Moen A-GG, Hansen TH, Nilssen KT (2005). Digestion of capelin, *Mallotus villosus* (Müller), herring, *Clupea harengus* L., and polar cod, *Boreogadus saida* (Lepichin), otoliths in a simulated seal stomach. ICES J. Mar. Sci..

[30] Yalden DW, Yalden PE (1985). An experimental investigation of examining Kestrel diet by pellet analysis. Bird Study.

[31] Llona ACP, Andrews PJ (1999). Amphibian taphonomy and its application to the fossil record of Dolina (middle Pleistocene, Atapuerca, Spain). Palaeogeogr., Palaeoclimatol., Palaeoecol..

[32] Uttendörfer O (1939). Die Ernährung der deutschen Raubvögel und Eulen und ihre Bedeutung in der heimischen Natur.

[33] Fernández-Jalvo Y (2016). Taphonomy for taxonomists: Implications of predation in small mammal studies. Quat. Sci. Rev..

[34] Comay, O., Weissbrod, L. & Dayan, T. Predictive modelling in paleoenvironmental reconstruction: The micromammals of Manot Cave, Israel. *J. Human Evol.* (In press). 10.1016/j.jhevol.2019.102652 31623862

[35] Fernández-Jalvo Y, Andrews P (1992). Small mammal taphonomy of Gran Dolina, Atapuerca (Burgos), Spain. J. Arch. Sci..

[36] Smith KT, Wuttke M (2015). Avian pellets from the late Oligocene of Enspel, Germany—ecological interactions in deep time. Palaeobiodiv. Palaeoenv..

[37] Rich PV, Bohaska DJ (1981). The Ogygoptyngidae, a new family of owls from the Paleocene of North America. Alcheringa.

[38] Myhrvold NP (2012). A call to search for fossilised gastric pellets. Hist. Biol..

[39] Farner, D. S. in *Biology and Comparative Physiology of Birds, Volume I* (ed Marshall, A. J.) 411–467 (Academic Press, 1960).

[40] Duke GE (1997). Gastrointestinal physiology and nutrition in wild birds. J. Nutr. Soc..

[41] O’Connor JK, Zhou Z-H (2020). The evolution of the modern avian digestive system: Insights from paravian fossils from teh Yanliao and Jehol Biotas. Palaeontology.

[42] Grimm RJ, Whitehouse WM (1963). Pellet formation in a Great Horned Owl: A roentgenographic study. Auk.

[43] Houston, D. C. & Duke, G. E. in *Raptor Research and Management Techniques* (eds Bildstein, K. L. & Bird, D. M.) 267–277 (Hancock House, 2007).

[44] Mennaga AMW (1938). Waterstofionenconcentratie en vertering in de maag van eenige vertebraten.

[45] Reed CI, Reed BP (1928). The mechanism of pellet formation in the Great Horned Owl (*Bubo virginianus*). Science.

[46] Chitty D (1938). A laboratory study of pellet formation in the short-eared Owl (*Asio flammeus*). Proc. Zool. Soc. Lond..

[47] Errington PL (1930). The pellet analysis method of raptor food habits study. Condor.

[48] Moon EL (1940). Notes on hawk and owl pellet formation and identification. Trans. Kansas Acad. Sci..

[49] Prum RO (2015). A comprehensive phylogeny of birds (Aves) using targeted next-generation DNA sequencing. Nature.

[50] Duke GE, Evanson OA, Redig PT, Rhoades DD (1976). Mechanism of pellet egestion in great-horned owls (*Bubo virginianus*). Am. J. Physiol..

[51] National Research Council (U.S.) (2006). Nutrient Requirements of Dogs and Cats.

[52] Christiansen JS, Gildberg A, Nilssen KT, Lindblom C, Haug T (2004). The gastric properties of free-ranging harp (*Pagophilus groenlandicus* (Erxleben, 1777)) and hooded (*Cystophora cristata*(Erxleben, 1777)) seals. ICES J. Mar. Sci..

[53] Friedman MHF (1939). Gastric secretion in birds. J. Cell. Comp. Physiol..

[54] LePrince P, Dandrifosse G, Schoffeniels E (1979). The digestive enzyme and acidity of the pellets regurgitated by raptors. Biochem. Syst. Ecol..

[55] Andrews P (1996). Experiments in taphonomy. J. Arch. Sci..

[56] Lyman RL (1994). Vertebrate Taphonomy.

[57] Brand LR, Hussey M, Taylor J (2003). Decay and disarticulation of small vertebrates in controlled experiments. J. Taphon..

[58] Christensen, R. H. B. Ordinal—regression models for ordinal data. https://cran.r-project.org/src/contrib/Archive/ordinal/ (R package version 2015.6-28) (2015).

[59] Hirk, R., Hornik, K. & Vana, L. Mvord: An R package for fitting multivariate ordinal regression models. https://cran.r-project.org/src/contrib/Archive/mvord/ (R package version 0.3.1) (2018).

[60] Comay, O. *Diet and Taphonomic Signatures of Owls as Tools for Paleoecological Reconstruction: Qesem Cave as a Test Case.* Ph.D. thesis, Tel Aviv University (2016).

[61] Gopher A, Barkai R (2016). State of the art at the multidisciplinary research at Middle Pleistocene Qesem Cave, Israel, 2015—An introduction. Quat. Intl..

[62] Smith, K. T., Maul, L. C., Flemming, F., Barkai, R. & Gopher, A. The microvertebrates of Qesem cave: A comparison of the two concentrations. *Quat. Intl.* **398**, 233–245 (2016).

[63] Comay, O. & Dayan, T. in *Themes in Old World Zooarchaeology: From the Mediterranean to the Atlantic* (eds Albarella, U. *et al.*) (Oxbow Books, In press).

[64] Le Tensorer, J.-M., von Falkenstein, V., Le Tensorer, H. & Muhesen, S. in *The Lower and Middle Palaeolithic in the Middle East and Neighbouring Regions (Études et Recherches Archéologiques de l’Université de Liège 126)* (eds Le Tensorer, J.-M., Jagher, R. & Otte, M.) 235–248 (Université de Liège, 2011).

