# Supplementary material for: A model of digestive tooth corrosion in lizards: experimental tests and taphonomic implications
Source: Sci Rep. 2021 Jun 18;11:12877. doi: 10.1038/s41598-021-92326-5 (PMC8213689; doi:10.1038/s41598-021-92326-5)
Supplement: Supplementary file 1 — Supplementary Information 1. [file 41598_2021_92326_MOESM1_ESM.pdf]

Supplementary Information for:

A model of digestive tooth corrosion in lizards: experimental tests and taphonomic implications

Krister T. Smith, Orr Comay, Lutz Maul, Fabio Wegmüller, Jean-Marie Le Tensorer, Tamar Dayan

Supplementary Note
Supplementary Figure S1
Supplementary Figure S2
Supplementary Figure S3
Supplementary Figure S4
Supplementary Figure S5
Supplementary Table S1
Supplementary Table S2
Supplementary Table S3
Supplementary Table S4
Supplementary Table S5
Supplementary Methods
Supplementary Data
Supplementary References

Supplementary Note

Bones or other mineralized parts of prey are found in the feces or intestine of sharks [1,2], actinopterygian fish [3], African Coelacanth [4], African [5] and Australian [6] lungfish, stem amphibians [7], mammals [8,9] including carnivorous bats [10,11], carnivorous squamates [12-15], carnivorous turtles [16-18], and crocodylians [19]. In these taxa, prey carcasses normally pass unidirectionally through the digestive tract. Similarly, coprolites indicate that bones were normally passed into the intestine in dinosaurs as far crownward as *Tyrannosaurus rex* [20], and none of the thousands of specimens of the dromaeosaur *Microraptor* preserves a pellet [21]. In contrast, pellets have been documented in the troodontid *Anchiornis* [21] and in enantiornithine birds [21-23], suggesting that this propensity arose on the branch separating Dromaeosauridae and Troodontidae + birds. Pellet production is of course well documented in numerous clades of extant birds.

In vertebrates, gastric acidity does not correlate well with proteolytic activity. For birds, Duke et al. [24] showed that diurnal raptors had relatively low gastric pH (1.3–1.8), whereas the two owls they monitored had pH of 2.2 and 2.5, closer to the pH in the gizzard of many other birds [25]. The Barn Owl appears to have the highest pH (lowest acidity), generally greater than 3.0 [26]. Proteolytic activity in the stomach, however, is high in both diurnal raptors and owls, as noted above. Carnivorous birds usually show higher gastric proteolytic activity than herbivorous birds [24,27-29].

Among mammals, published data on even zoo animals are surprisingly meager; data on morbid or experimental animals are easier to come by. Normal beagle dogs had an average baseline pH of 1.8 [30] or less [31]. Christiansen et al. [32], while noting large differences among animals taken at random (with regard to feeding state), suggested that a stomach pH of about 2.0 was necessary for adequate digestion in their two seal species. Data in Fox et al. [33] suggest that a pH between 1.5 and 2.0 is normal for the Ferret. On the other hand, domestic cats are reported to have relatively high pH [2.5; ascribed to Brosey et al. (2000) by National Research Council (U.S.) [34]]. Proteolytic activity in the stomach, as noted above, is low in examined carnivoran mammals, including those that bracket Ferrets phylogenetically.

Intestinal reflux has been documented in a wide variety of extant birds, including members of both Palaeognathae and Neognathae [35].

Supplementary Figure S1

Micro-computed tomography reconstruction of a cheek tooth of the pleurodont lizard *Iguana iguana* in transverse section. The enamel (white) is confined to the tooth tip, whereas the rest of the tooth comprises a ‘dentine cone’. Courtesy: M. Wirkner (Senckenberg Research Institute) and T. Tütken (University of Mainz).

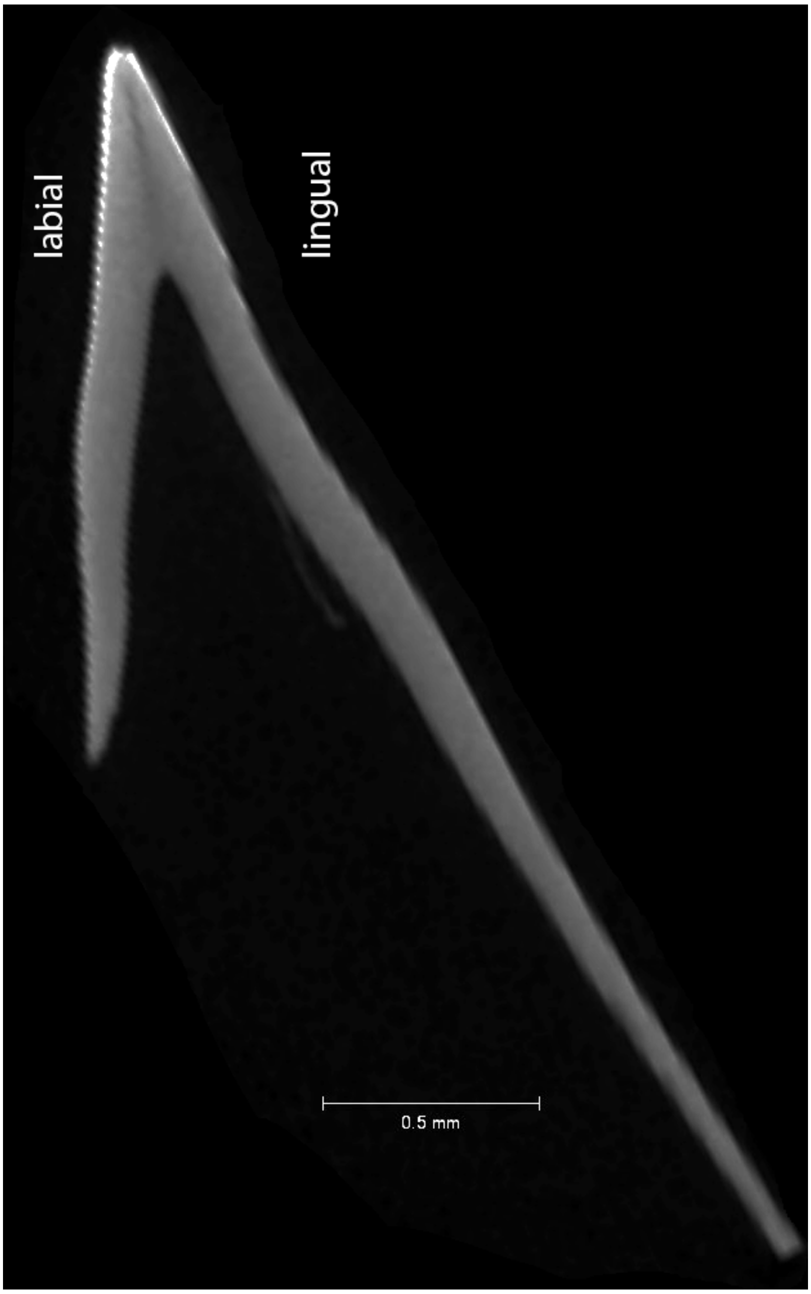

Supplementary Figure S2

Corrosion induced on teeth of Green Anoles (*Anolis carolinensis*) by artificial gastric solutions (Supplementary Table S1). (**a-d**) whole lower jaw, whole teeth, and close-ups of crown and mid-shaft on specimen #2 (control); (**e-f**) whole lower jaw and whole teeth of specimen #8; (**g-j**) whole lower jaw, whole teeth, and close-ups of crown and mid-shaft on specimen #25; (**k-l**) whole lower jaw and whole teeth of specimen #26; (**m**) whole lower jaw of specimen #24; (**n-q**) whole lower jaw, whole teeth and close-ups of crown and mid-shaft of specimen #12; (**r-v**) whole lower jaw, whole teeth and close-ups of crown, upper shaft and lower shaft on specimen #32; (**w**) whole lower jaw of specimen #29. Scale bars: left panels 1 mm, middle panels 300 μm, right panels 10 μm.

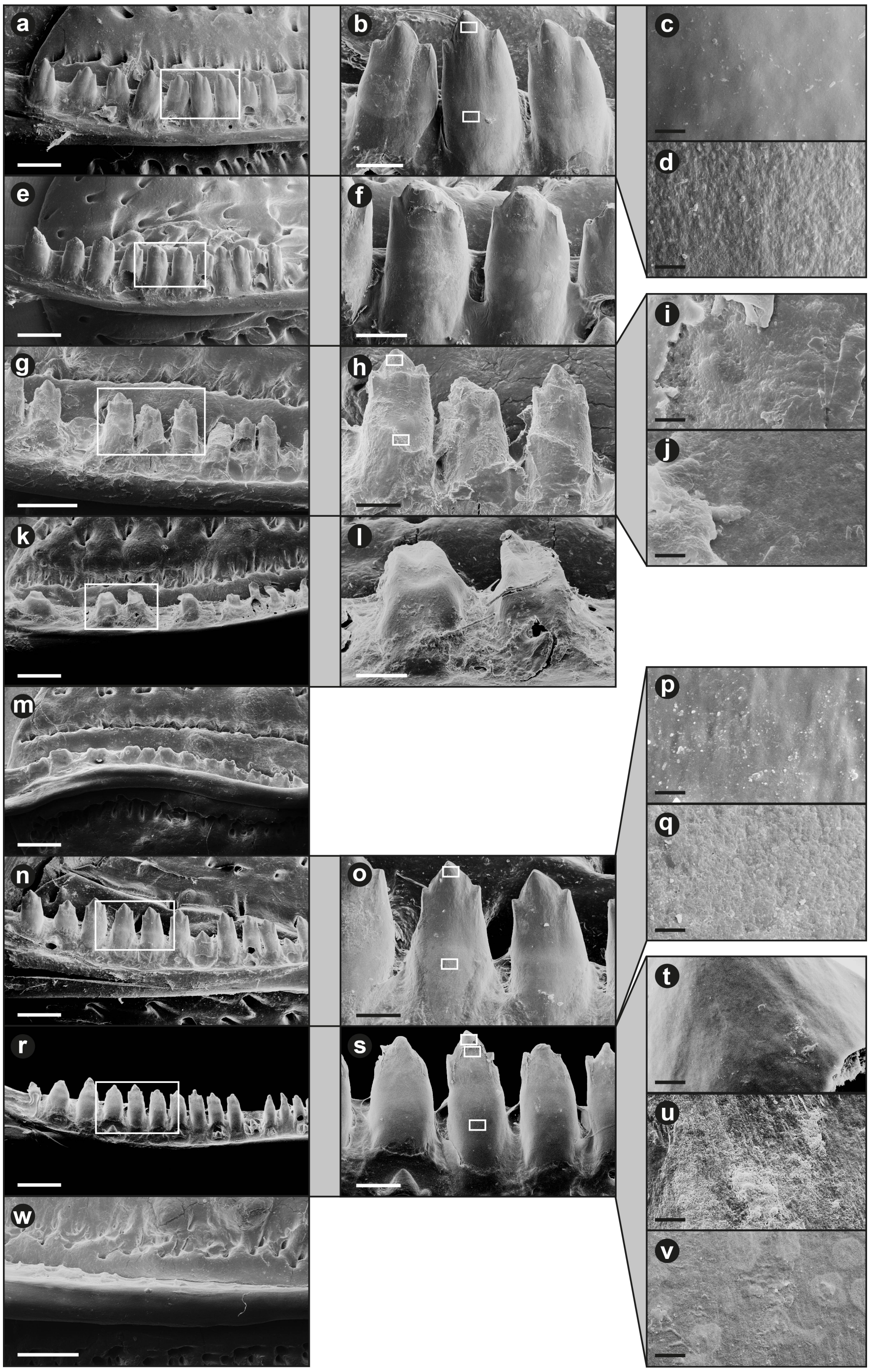

Supplementary Figure S3

Relationship between type 1 and type 2 corrosion patterns (symbols correspond to those in Fig. 1), where they occur ($N = 20$), and acid-enzyme conditions in the experiments with simulated gastric fluids. Where two specimens would plot at the same position, the symbols are separated and the position indicated with arrows. Type 2 corrosion is concentrated in the low-acid (pH ≥ 2.5), high-enzyme (pepsin concentration ≥ 3.0 mg/ml) area of parameter space. Type 1 corrosion occurs more broadly but is the only pattern found at high-acid (pH ≤ 2.5), low-enzyme (pepsin concentration ≤ 3.0 mg/ml) area of parameter space. Where type 1 corrosion occurs in the low-acid, high-enzyme area, it is after very long exposure times (20 h).

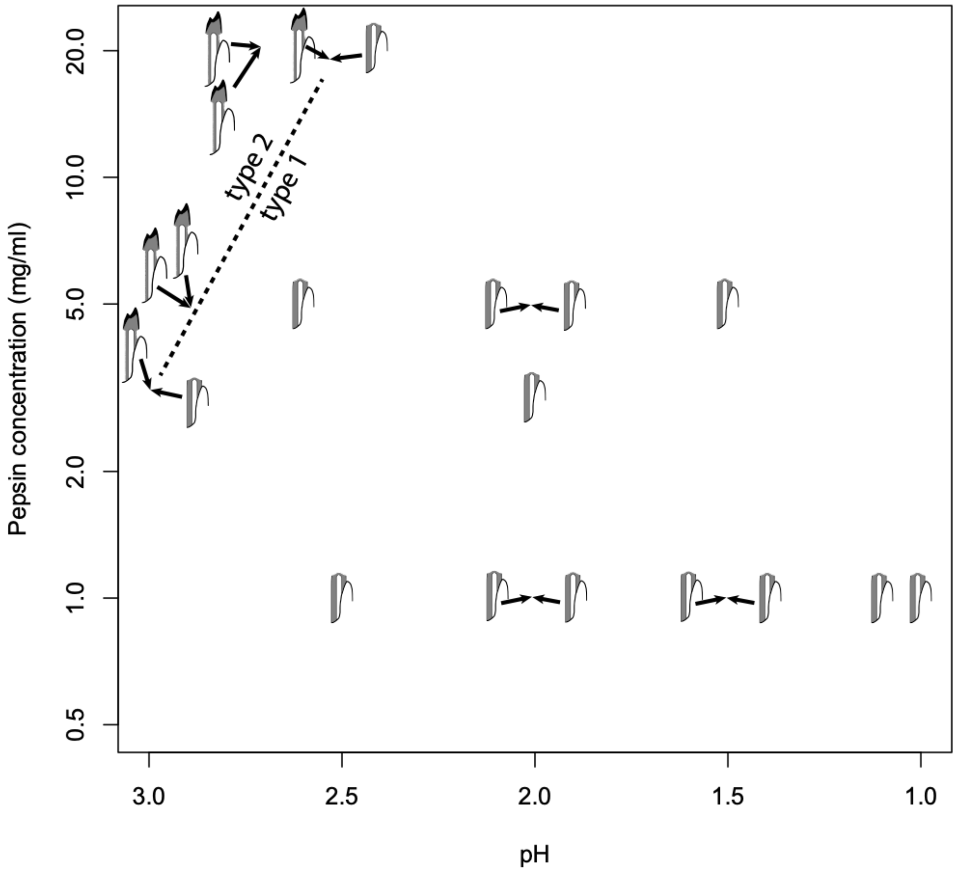

Supplementary Figure S4

Corrosion damage to teeth of lizards ingested by a Barn Owl (*Tyto alba*). (**a**) whole lower jaw and close-up of Bridled Skink (*Trachylepis vittata*) from pellet #1239; (**b**) whole lower jaw and close-up of undetermined gecko from pellet #652. This “type 3” pattern of damage, in which the neck between the crown and shaft is preferentially corroded was not predicted by the model. Scale bars: whole jaws, 1 mm; close-ups, 0.5 mm.

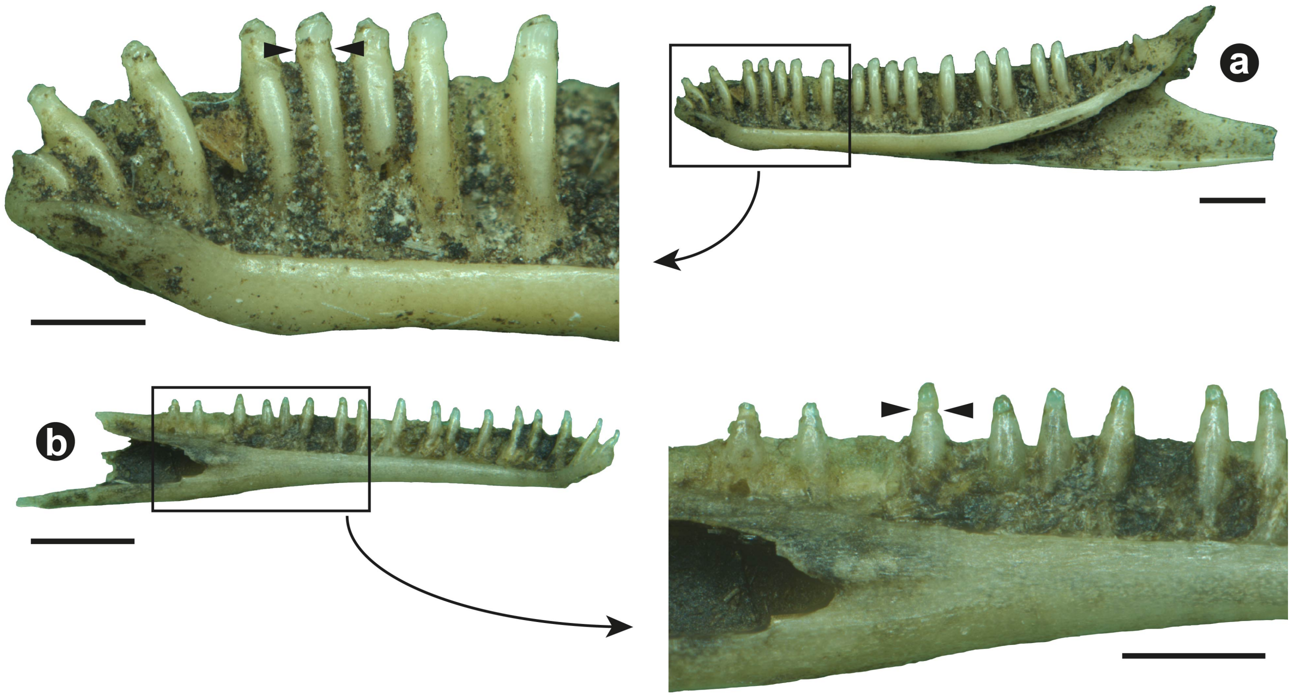

Supplementary Figure S5

Digestive processes in gnathostomes (jawed vertebrates). (**a**) Digestion in a generic gnathostome in relation to the discrete portions of the digestive tract. (**b**) In enteric digesters, the plesiomorphic strategy maintained in Carnivora, prey is fully digested only in the intestine, after corrosion from gastric acid has ceased. (**c**) In gastric digesters such as raptors, prey is digested in the stomach before the indigestible parts are ejected through the mouth. The asterisk indicates the place where most de-fleshing takes place.

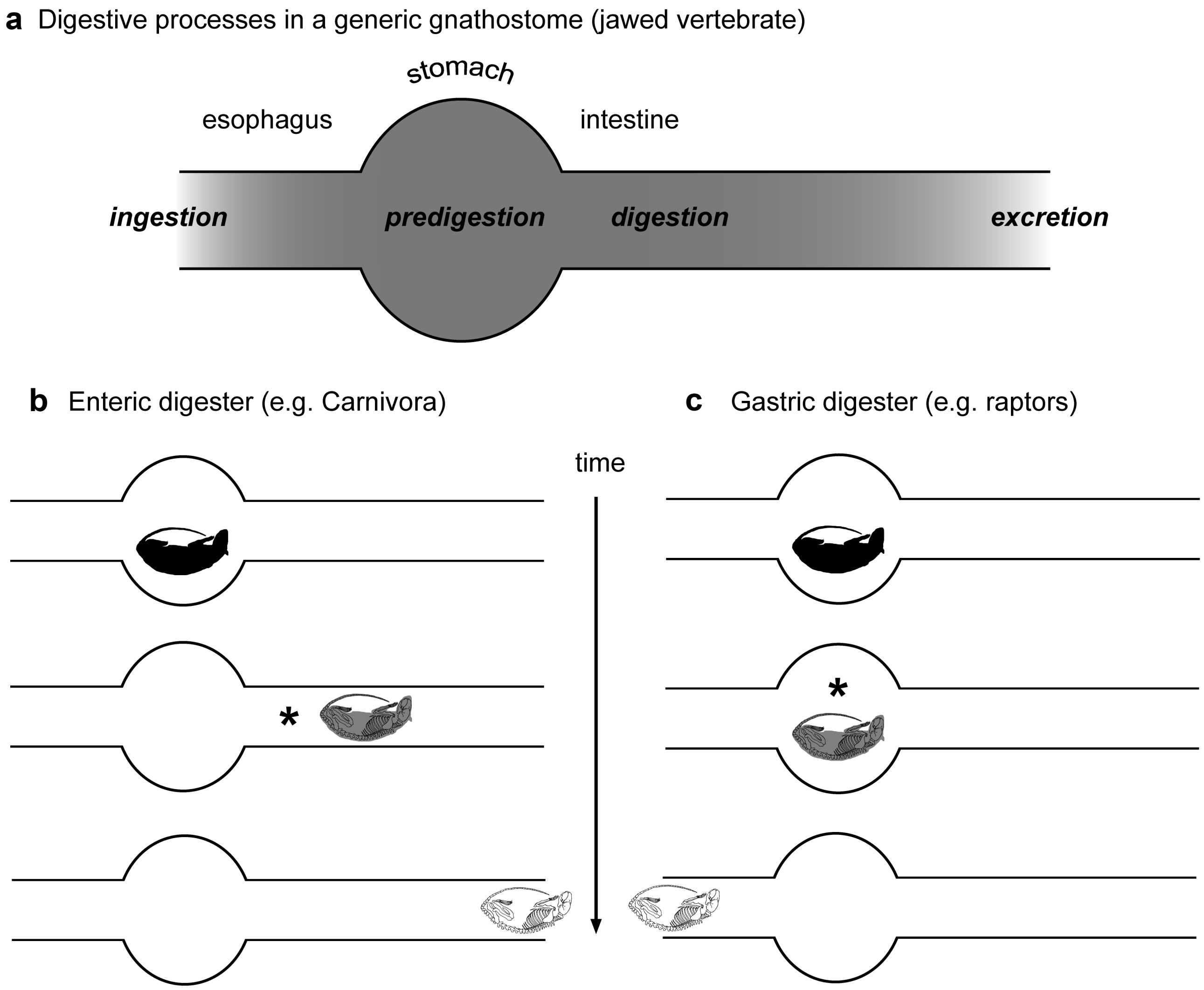

Supplementary Table S1

Carcass Nr.	Initial pH	Pepsin concen-tration (mg/ml)	Duration (h)	Lizard size: SVL (mm)	Lizard size: Mass (g)	Additional experimental procedures	Modern predator analog
2.	7.65	n/a	168	55	4.80	macerated	(control)
1.	n/a	n/a	n/a	62	5.37	dermestids	(control)
27.	1.0	1	3	61	4.73		
23.	1.0	1	18.5	55	3.60		
24.	1.1	1	4	60	5.26		carnivoran
25.	1.2	1	2	61	5.22		carnivoran
21.	1.5	1	2	67	4.92		carnivoran
26.	1.5	1	4	62	5.25		carnivoran
28.	1.5	1	4	57	4.71	mouth closed	carnivoran
22.	1.5	5	2	57	4.06		
29.	1.5	5	5	60	4.49		diurnal raptor
15.	2.0	1	5	55	3.92		
18.	2.0	1	10	58	5.14		
16.	2.0	3	5	66	7.05		
19.	2.0	3	10	58	5.02		
17.	2.0	5	5	69	8.06		
20.	2.0	5	10	61	4.87		
13.	2.4	5	20	61	3.34		
3.	2.5	1	20	62	6.36		
5.	2.5	20	20	57	4.50	dismembered, shaken	
6.	2.5	20	20	58	4.26		
30.	2.6	5	20	58	4.00	shaken	
31.	2.7	20	10	58	4.52	dismembered, shaken	
32.	2.7	20	10	49	2.29	shaken	
33.	2.7	20	10	48	2.47	dismembered	

34.	2.7	20	10	42	1.66	dismembered, shaken	
35.	2.7	20	10	42	1.93	shaken	
12.	2.9	5	10	62	4.91		owl
7.	2.9	1	10	55	3.02		
9.	2.9	3	10	55	2.93		
11.	2.9	5	5	62	3.95		owl
8.	3.0	1	20	59	4.89		
4.	3.0	3	20	58	5.24		
10.	3.0	3	20	62	6.03		
14.	3.0	5	20	57	4.99		owl

Experimental treatments on carcasses of *Anolis carolinensis* using artificial gastric fluids, ordered first by initial pH, then pepsin concentration, then duration. Body size given as snout-vent length (SVL) and mass.

Supplementary Table S2

Pellet Nr.	Owl species	Site	Lizard taxon	Lizard element
462	*Tyto alba*	Barza Crater	*Trachylepis vittata*	L+R dentary, L maxilla
652	*Tyto alba*	Old Mifratz	Gekkota indet.	L dentary, L+R maxilla
712	*Athene noctua*	Iris Hill, Rishon LeTzion	Scincidae indet.	L maxilla
810	*Tyto alba*	Gaf Stream	Gekkota indet.	L+R dentary, L+R maxilla
977	*Asio otus*	Neve Yaraq	Lacertidae indet.	R maxilla
1239	*Tyto alba*	Zikhron Yaakov	*Trachylepis vittata*	R dentary

Provenance of owl pellets from which modern pleurodont lizard jaws were extracted. All were collected in Israel as part of the Ph.D. dissertation of Comay [36]. Abbreviations: R = right, L = left.

Supplementary Table S3

**Locality name**	**Age**	**Location**	**Setting**	***N***
Qesem Cave	Middle Pleistocene	Israel	Cave	18
Hummal Layer G	Early or Middle Pleistocene	Syria	Open air, mudstone	33
Big Multi	Late Paleocene	Wyoming, USA	Mudstone, weak paleosol	55
Castle Gardens	Early Eocene	Wyoming, USA	Clayey sandstone, scour-fill	145
Dorsey Creek	Early Eocene	Wyoming, USA	Crevasse splay, weak paleosol	91
Level M	Early Eocene	Wyoming, USA	Siltstone and channel sandstone	154
Level O	Early Eocene	Wyoming, USA	Carbonaceous mudstone	146
Level S	Early Eocene	Wyoming, USA	Carbonaceous mudstone	42
Turtle Graveyard	Early Eocene	Wyoming, USA	Limestone, calcareous mudstone	207

Pleistocene and early Paleogene fossil sites from which pleurodont lizard jaws were examined. *N* is the number of such jaws from each site. References on setting and lizard content: Qesem Cave [37-39], Hummal [40,41], Big Multi [42] (lizards in prep), Castle Gardens [43,44], Dorsey Creek Quarry [45,46], and Bitter Creek localities (Levels M, O, and S and Turtle Graveyard) [47-50]. The Paleogene sites represent all the late Paleocene and early Eocene sites studied by the respective authors, not a selection thereof.

Supplementary Table S4

	N	***n* showing Corrosion (C) to crown**	***n* showing Corrosion (C) to shaft**	**C(crown) > C(shaft)**	**C(shaft) > C(crown)**
High-acid, low-enzyme end-member	5	4	3	4	0
Low-acid, high-enzyme end-member	3	1	3	1	2

Summary of corrosion damage to teeth of *Anolis carolinensis* exposed to artificial gastric fluids (without special treatments like shaking). Among them, five corresponded to one physiological end-member (high-acid, low-enzyme), and three corresponded to the other physiological end-member (low-acid, high-enzyme). The missing specimen in the right two columns for the first end-member showed approximately equal damage to crown and shaft.

Supplementary Table S5

Owl species	*N*	*n* showing Corrosion (C) to crown	*n* showing Corrosion (C) to shaft	C(crown) > C(shaft)	C(shaft) > C(crown)
Asio otus	1	0	0	NA	NA
Athene noctua	1	0	0	NA	NA
Tyto alba	4	(2)	0	2	0
**TOTALS**	**6**	**(2)**	**0**	**2**	**0**

Summary of corrosion damage to teeth of pleurodont lizards extracted from owl pellets. The parentheses indicate that while present, the corrosion was restricted to a very small number of the teeth in one of the jaws of the individual.

Supplementary Methods

As a control for the experiments, one anole carcass was macerated in 50 ml of de-ionized water (initially, pH = 7.0) for 7 days. Because even in this pure-water control treatment the fluid chemistry will change as a result of decomposition, a second control was prepared using dermestid beetles without exposure to any fluid. Controls were selected at random.

Our conceptual framework has two active components, HCl and enzymes. For experimental purposes, the enzyme component was represented by pepsin, the main digestive enzyme in the stomach [51]. Swine pepsin (pepsin A) was used (Fagron GmbH, Item No. 197140). The activity of swine pepsin on many substrates dramatically decreases when pH rises above 2.5–3.0 [52,53]. The activity peaks of various digestive enzymes with respect to temperature are relatively broad compared to the body temperature differences exhibited by homeothermic vertebrates (birds, placental mammals) [54: fig. 5-24]. Thus, we do not expect that temperature will have any significant influence on the interpretation of the experiments. Pepsin efficacy was tested on chicken muscle immediately prior to the beginning of the experiment, whereby mass reductions of up to 50% were observed over about 4 hr.

Fresh carcasses of (sub)adult anoles were exposed to a variety of simulated gastric fluids consisting of HCl and pepsin mixed with distilled water to form 30–50 ml of solution (Supplementary Table S1), the minimum required to cover a carcass fully. Specimens had a restricted size range of 49–69 mm (snout-vent length), corresponding to a mass of 2.29–8.06 g. These solutions sampled many combinations of pepsin concentration (1.0, 3.0, 5.0, and 20 mg pepsin/ml solution) and initial pH (1.0–3.0). Exposure times of carcasses to the fluid varied (≤5, 10 or 20 hr). The mouth of most specimens was propped open with a narrow plastic strip to expose teeth to the fluids, because inital study of a carcass (#28) with closed mouth showed no corrosion at all.

The experimental parameters (pH, pepsin concentration, duration) were calibrated with natural predators in mind (Supplementary Table S1). Experimental treatments of short duration (<4 hr) under high-acid (pH ≤ 2.0), low-pepsin (≤ 3 mg/ml fluid) conditions were meant to mimic mammalian carnivores, which represent the high-acid, low-enzyme physiologic end-member. Gastric emptying times are the relevant expression of the duration of predigestion, since pH rises toward neutrality and pepsin activity ceases in the intestine. Half-emptying times vary greatly in dogs [34], partly in dependence on diet, although it averages ~3 hr, with total emptying times of 4–6 hr [55]. For cats, a wider range has been documented for half-emptying times [34], but durations of 4–6 hr also seem typical [56]. Although little data is available on wild or other carnivores, and both dogs and cats are domesticated species, they phylogenetically bracket the clade Carnivora [57,58].

Experimental treatments of long duration (10–20 hr) under low-acid (pH ≈ 2.5), high-pepsin (≥ 5 mg/ml) conditions were meant to mimic owls, which represent the low-acid, high-enzyme physiologic end-member. Meal-to-pellet intervals (MPI), equivalent to gastric emptying times for mammalian predators, are much longer. Under a variety of conditions, MPI in examined owls species was usually >10 hr [59] and frequently up to 20 hr [60,61]. It may also be noted that in diurnal raptors, MPI of around 20 hr is the norm [24,60,62]. Thus, although MPI varies up to twofold among birds of prey, gastric residence time is always long compared to mammalian carnivores. This characteristic appears to be apomorphic of raptors, given the very short passage times for food in the digestive tract seen in many frugivorous and insectivorous members of Neoaves and Galloanseres [63].

The pH was measured using an electronic pH-meter (Hanna Instruments model HI 98103, with a nominal precision of 0.01) at the beginning and end of each experimental treatment. Most treatments were conducted in loosely lidded glass flasks placed in a desiccation chamber heated to a constant temperature of 40ºC. Most carcasses were placed whole into the treatment fluids. For the desiccation chamber, test runs with water heated to 40ºC for the maximum duration of the experiment were conducted to ensure that evaporation, which could influence water chemistry, was negligible. After completion, the simulated gastric fluid was neutralized and flushed, and the lower jaws were excised and preserved.

Eight specimens were subjected to more extreme conditions to study other parameters. Seven of these were placed in solutions with a pepsin concentration of 20 mg/ml (Supplementary Table S1), considerably higher than has been documented in living predators. Four were cut into three pieces (at the neck and anterior to the pelvis) to simulate prey dismemberment. The flasks of six specimens were placed in a shaking water bath at an intentionally extreme rate of 120 revolutions per minute (rpm), where the temperature of the water bath was set to 40ºC, to simulate strong gastric motility. Notably, however, gastric motility is considered minor in owls, which is consistent with their weak gizzard walls [63,64].

One dentary from each experimental and control specimen was extracted and studied using a binocular microscope. They were then affixed to stubs, coated in a gold-paladium alloy, and examined using a scanning electron microscope (SEM) at the Senckenberg Research Institute in Frankfurt. Qualitatively, we assessed whether the corrosion patterns corresponded to the predicted patterns, type 1 or type 2, and whether any other, unpredicted patterns emerged. Quantitatively, we counted in how many specimens corrosion was greater to the crown of the teeth than the shaft, and vice versa, as this anatomical distinction informed the conceptual model.

Supplementary Data

A gradual rise in the pH was observed over the course of all treatments in the laboratory experiments. Especially for high-pH, extended-time treatments, these results and published activity curves for swine pepsin suggest that it would have become inactive later in the treatments. However, since pH units are logarithmic, the pepsin may have still been active for most of the course of each treatment, not a small fraction.

Generally speaking, the poor digestion of the carcasses by the pepsin even at extremely high (biologically unrealistic) concentrations is noteworthy. The experimental specimens remained intact after exposure to the simulated gastric fluids. In some cases, the tongue, oral mucosa, throat and body wall exhibited extensive degradation. In one dismembered specimen that was weighed also at the end of the treatment, carcass mass was considerably reduced (by 35%), indicating that direct access to the body cavities aided the efficacy of the pepsin. Still, it is clear that mere exposure of a lizard carcass, intact or not, to pepsin will be insufficient to de-flesh it completely before the bones would be ejected as a pellet. This result suggests the importance of post-gastric enzymes for digestions in bird of prey.

The enamel surface of both the crown and shaft is smooth in the control specimens (Supplementary Fig. S2a–d). Deep on the shaft, the surface is smooth or has a fine-scale texture consisting of low, rounded mounds (Supplementary Fig. S2d).

High-acid, low-enzyme solutions

Solutions representing the high-acid, low-enzyme end-member covered pH ≤ 1.5 and pepsin concentrations of 1 mg/ml (Supplementary Table S1). These solutions produced significant corrosion to the lizard teeth, especially to the crowns. In many cases where specimens were exposed to these conditions for 2 hr or more (#21, 22, 24, 26, 27), the crowns of the teeth above the gumline were completely decalcified, leaving at most the brown pliable protein matrix behind (Supplementary Fig. S2k–l). This supports the hypothesis that the gingiva plays a protective role against corrosion of the tooth shaft by acid. As the cusps are principally composed of enamel, little crown morphology remained. The shafts of the same teeth, while sometimes appearing slightly corroded, are always much better preserved than the crowns. One specimen (#25), exposed to a solution with pH = 1.2 and pepsin = 1 mg/ml for 2 hr, produced considerable corrosion to the tooth crowns, which had been stripped away in patches (Supplementary Fig. S2g–j).

Carcasses spending more than a short time in a very low-pH (~1.0) solution were heavily corroded, as expected given the findings of Fernández-Jalvo and Andrews [65]. Even 3 hr at such low

pH was sufficient to decalcify the bones and teeth (remove the hydroxyapatite), leaving a brown, pliable structure probably made of a collagen matrix that curled upon drying (#24, 27). In a specimen (#23) that spent 18.5 hr in a pH = 1.0 solution, the mineral substance of the entire skeleton was destroyed (Supplementary Fig. S2m).

Corrosion to the teeth that closely corresponds to the prediction "type 1" was observed in three (#21, 24, 26) of the five specimens specifically considered to represent the physiological end-member (Supplementary Fig. S2k–l; Supplementary Table S1). Such corrosion was also observed in 11 other specimens, especially in the high-acid, low-enzyme area of parameter space. Note that we consider specimens in which the crown has been completely decalcified as representing type 1 corrosion, even if the protein matrix remains, because the matrix would quickly be destroyed in the diagenetic environment [9,19]. More generally, corrosion damage to the crown was greater than that to the shaft in four specimens, whereas the reverse was true in no specimen (Supplementary Table S4).

Low-acid, high-enzyme solutions

Solutions representing the low-acid, high-enzyme physiological end-member covered pH ≈ 3.0 and pepsin concentrations = 5 mg/ml (Supplementary Table S1). The digestion was also allowed generally allowed to proceed for a longer time (10 or 20 hr). These solutions produced corrosion on the teeth of all of the specimens (Supplementary Table S4), but the distribution of the corrosion was different. In two of the specimens (#11, 12), the shaft was corroded but the crown was unaffected (Supplementary Fig. S2n–q).

Corrosion roughly corresponding with type 2 was induced on a total of 6 specimens, all of which are concentrated in the low-acid, high-enzyme area of parameter space (Supplementary Fig. S3). Three of these were subjected to special treatments like shaking, however (Supplementary Table S1), and so they do not appear in Supplementary Table S4. Fisher's exact test on the last two columns in Supplementary Table S4 yields a non-significant *P*-value of 0.143; according to this conservative test, the end-members cannot be satisfactorily distinguished, which could be explained by low sample size or the lack of an effect. However, multiple logistic regression of pH, pepsin concentration and duration on all treatments (N=20) producing approximations of type 1 and type 2 corrosion (Supplementary Fig. S2) yields positive (favoring type 2 over type 1) regression coefficients with significant *P*-values for pH (P = 0.00028) and pepsin concentration (P = 0.024), as predicted by the model.

More extreme conditions (pH = 2.5–2.7, pepsin concentrations 20 mg/ml), produced greater corrosion than for specimens #11 and 12 that nevertheless closely corresponded to type 2 corrosion.

In particular, specimens #5, 32 and 33 showed considerable corrosion of the shaft that extended up onto the crown (Supplementary Fig. S2r–v). The enamel was partly removed from the crown, but apically (around the cusps) it is preserved and uncorroded. Thus, a strong step is present between uncorroded enamel and corroded dentine. Specimen #34 shows greater damage to the crown than the shaft. By comparison with #32 and 33, it is likely that the enamel had completely spalled off the crown, such that corrosive damage to the crown exceeded that to the shaft.

High-acid, high-enzyme solutions

It is expected that, since both acid and pepsin are known to corrode bony tissues, high-acid, high-enzyme conditions will produce greater corrosion than either of the end-members examined above. The teeth of three specimens (e.g., #19) exposed to such conditions were completely destroyed down to the bone, which does not itself appear corroded to the naked eye (Supplementary Fig. S2w). No other specimen did. It is likely that once the pulp cavity of the tooth has been breached, corrosive fluid may penetrate to the base of the tooth and destroy it from the inside without significantly affecting the tooth-bearing bone.

Low-acid, low-enzyme solutions

Only two treatments used low-acid, low-enzyme solutions: #7 and #8 (Supplementary Table S1). The latter ran for 20 hr. Here, the enamel on the crown above the gumline is distinctly eroded in a shallow patch at the lingual base of the central cusp between the accessory cusps (Supplementary Fig. S2e–f). The former, which ran for half the time (10 hr), shows a more advanced state of the same corrosion, where the corroded patches have expanded over much of the crown. In some places the enamel caps of the cusps have been entirely destroyed. In neither case do we see broad surfaces of enamel where corrosion is indicated by fine pitting over a large area; rather, corrosion is more discrete. Corrosion to the shaft is not seen in either.

Influence of dismemberment and shaking

It was expected that shaking would increase fluid access to the reaction areas on the tooth or gingiva and so would lead to greater corrosion. Three specimens (#33-35) were subjected to shaking and/or dismemberment but otherwise identical conditions: pH = 2.7, pepsin concentration 20 mg/ml, duration 10 hr (Supplementary Table S1). The specimen that was merely dismembered but not shaken (#33) indeed showed considerably less total corrosion than the specimens that were

shaken, or dismembered and shaken. Due to low sample sizes, no definite conclusions can be drawn about the influence of shaking (and hence gastric motility).

Bibliography

1 Stewart, J. D. Enterospirae (fossil intestines) from the Upper Cretaceous Niobrara Formation of western Kansas. *Univ. Kansas Paleont. Contrib.* **89**, 9-16 (1978).

2 Williams, M. E. The origin of "spiral coprolites". *Univ. Kansas Paleont. Contrib.* **59** (1972).

3 Wilson, M. V. H. Predation as a source of fish fossils in Eocene lake sediments. *Palaios* **2**, 497-504 (1987).

4 Uyeno, T. & Tsutsumi, T. Stomach contents of *Latimeria chalumnae* and further notes on its feeding habits. *Environ. Biol. Fishes* **32**, 275-279 (1991).

5 Corbet, P. S. The food of non-cichlid fishes in the Lake Victoria Basin, with remarks on their evolution and adaptation to lacustrine conditions. *Proceedings of the Zoological Society of London* **136**, 1-101 (1961).

6 Kemp, A., Anderson, T., Tomley, A. & Johnson, I. in *Proceedings of the Sixth Australian Weeds Conference, Volume I* 155-158 (CSIRO, 1981).

7 Northwood, C. Early Triassic coprolites from Australia and their palaeobiological significance. *Palaeontol.* **48**, 49-68 (2005).

8 Andrews, P. & Evans, E. M. N. Small mammal bone accumulations produced by mammalian carnivores. *Paleobiology* **9**, 289-307 (1983).

9 Fisher, D. C. Taphonomic interpretation of enamel-less teeth in the Shotgun local fauna (Paleocene, Wyoming). *Contributions from the Museum of Paleontology, the University of Michigan* **25**, 259-275 (1981).

10 Bonato, V., Gomes Facure, K. & Uieda, W. Food habits of bats of subfamily Vampyrinae in Brazil. *Journal of Mammalogy* **85**, 708-713 (2004).

11 Medellín, R. A. Prey of *Chrotopterus auritus*, with notes on feeding behavior. *Journal of Mammalogy* **69**, 841-844 (1988).

12 Auffenberg, W. *The Behavioral Ecology of the Komodo Monitor*. (University Presses of Florida, 1981).

13 Auffenberg, W. *The Bengal Monitor*. (University Press of Florida, 1994).

14 Blain, A. W. & Campbell, K. N. A study of digestive phenomena in snakes with the aid of Roentgen ray. *Am. J. Roentgenol. Rad. Ther.* **48**, 229-239 (1942).

15 Stanner, M. & Mendelssohn, H. The diet of *Varanus griseus* in the southern coastal plain of Israel (Reptilia: Sauria). *Israel J. Zool.* **34**, 67-75 (1986/1987).

16 Demuth, J. P. & Buhlmann, K. A. Diet of the turtle *Deirochelys reticularia* on the Savannah River Site, South Carolina. *Journal of Herpetology* **31**, 450-453 (1997).

17 Kear, B. P. First gut contents in a Cretaceous sea turtle. *Biol. Lett.* **2**, 113-115 (2006).

18 Sung, Y.-H., Hau, B. C. H. & Karraker, N. E. Diet of the endangered big-headed turtle *Platysternon megacephalum*. *PeerJ* **4**, e2784 (2016).

19 Fisher, D. C. Crocodilian scatology, microvertebrate concentrations, and enamel-less teeth. *Paleobiology* **7**, 262-275 (1981).

20 Chin, K., Tokaryk, T. T., Erickson, G. M. & Calk, L. C. A king-sized theropod coprolite. *Nature* **393**, 680-682 (1998).

21 O'Connor, J. K. & Zhou, Z.-H. The evolution of the modern avian digestive system: insights from paravian fossils from the Yanliao and Jehol Biotas. *Palaeontol.* **63**, 13-27 (2020).

22 Dalsätt, J., Zhou, Z., Zhang, F. & Ericson, P. G. P. Food remains in *Confuciusornis sanctus* suggest a fish diet. *Naturwissenschaften* **93**, 444-446 (2006).

23 Wang, M., Zhou, Z.-H. & Sullivan, C. A fish-eating enantiornithine bird from the Early Cretaceous of China provides evidence of modern avian digestive features. *Current Biology* **26**, 1170-1176 (2016).

24 Duke, G. E., Jegers, A. A., Loff, G. & Evanson, O. A. Gastric digestion in some raptors. *Comparative Biochemistry and Physiology* **50A**, 649-656 (1975).

25 Farner, D. S. The hydrogen ion concentration in avian digestive tracts. *Poultry Sci.* **21**, 445-450 (1942).

26 Smith, C. R. & Richmond, M. E. Factors influencing pellet egestion and gastric pH in the Barn Owl. *Wilson Bull.* **84**, 179-186 (1972).

27 Herpol, C. Activité protéolytique de l'appareil gastrique d´oiseaux granivores et carnivores. *Ann. Biol. anim. Bioch. Biophys.* **4**, 239-244 (1964).

28 Herpol, C. Étude de l'activité protéolytique des divers organes du système digestif de quelques espèces däoiseaux en rapport avec leur régime alimentaire. *Z. vergl. Physiol.* **57**, 209-217 (1967).

29 Jain, D. K. Histomorphology and proteolytic activity in the gastric apparatus of frugivorous, carnivorous and omnivorous species of birds. *Acta biol. Acad. Sci. hung.* **27**, 135-145 (1976).

30 Lui, C. Y. *et al.* Comparison of gastrointestinal pH in dogs and humans: implications on the use of the beagle dog as a model for oral absorption in humans. *J. Pharm. Sci.* **75**, 271-274 (1986).
31 Youngberg, C., Wlodyga, J., Schmaltz, S. & Dressman, J. B. Radiotelemetric determination of gastrointestinal pH in four healthy beagles. *Am. J. Vet. Res.* **46**, 1516-1521 (1985).
32 Christiansen, J. S., Gildberg, A., Nilssen, K. T., Lindblom, C. & Haug, T. The gastric properties of free-ranging harp (*Pagophilus groenlandicus* (Erxleben, 1777)) and hooded (*Cystophora cristata* (Erxleben, 1777)) seals. *ICES J. Marine Sci.* **61**, 287-292 (2004).
33 Fox, J. G., Otto, G., Taylor, N. S., Rosenblad, W. & Murphy, J. C. *Helicobacter mustelae*-induced gastritis and elevated gastric pH in the Ferret (*Mustela putorius furo*). *Infect. Immun.* **59**, 1875-1880 (1991).
34 National Research Council (U.S.). *Nutrient Requirements of Dogs and Cats*. (National Academies Press, 2006).
35 Duke, G. E. Gastrointestinal physiology and nutrition in wild birds. *J. Nutr. Soc.* **56**, 1049-1056 (1997).
36 Comay, O. *Diet and taphonomic signatures of owls as tools for paleoecological reconstruction: Qesem Cave as a test case* Ph.D. thesis, Tel Aviv University, (2016).
37 Maul, L. C. *et al.* Microfaunal remains at Middle Pleistocene Qesem Cave, Israel: preliminary results on small vertebrates, environment and biostratigraphy. *Journal of Human Evolution* **60**, 464-480 (2011).
38 Gopher, A. & Barkai, R. State of the art at the multidisciplinary research at Middle Pleistocene Qesem Cave, Israel, 2015 - An introduction. *Quat. Intl.* **398**, 1-5 (2016).
39 Smith, K. T., Maul, L. C., Flemming, F., Barkai, R. & Gopher, A. The microvertebrates of Qesem Cave: a comparison of the two concentrations. *Quat. Intl.* **398**, 233-245 (2016).
40 Ismail-Meyer, K. Reconstruction of some site formation processes of Hummal (Syria). *Frankfurt. geowiss. Arb.* **30** (2009).
41 Maul, L. C. *et al.* Microvertebrates from unit G/layer 17 of the archaeological site of Hummal (El Kowm, Central Syria): preliminary results. *L'anthropol.* **119**, 676-686 (2015).
42 Wilf, P., Beard, K. C., Davies-Vollum, K. S. & Norejko, J. W. Portrait of a late Paleocene (early Clarkforkian) terrestrial ecosystem: Big Multi Quarry and associated strata, Washakie Basin, southwestern Wyoming. *Palaios* **13**, 514-532 (1998).
43 Yans, J. *et al.* High-resolution carbon isotope stratigraphy and mammalian faunal change at the Paleocene-Eocene boundary in the Honeycombs area of the southern Bighorn Basin, Wyoming. *American Journal of Science* **306**, 712-735 (2006).
44 Smith, K. T. A new lizard assemblage from the earliest Eocene (zone Wa0) of the Bighorn Basin, Wyoming, USA: Biogeography during the warmest interval of the Cenozoic. *Journal of Systematic Palaeontology* **7**, 299-358 (2009).
45 Silcox, M. T. & Rose, K. D. in *Eocene Biodiversity: Unusual Occurrences and Rarely Sampled Habitats* (ed G. F. Gunnell) 131-164 (Kluwer Academic/Plenum Publishers, 2001).
46 Smith, K. T. The long-term history of dispersal among lizards in the early Eocene: New evidence from a microvertebrate assemblage in the Bighorn Basin of Wyoming, USA. *Palaeontol.* **54**, 1243-1270 (2011).
47 Savage, D. E., Waters, B. T. & Hutchison, J. H. in *Field Conference on Tertiary Biostratigraphy of Southern and Western Wyoming, August 5-10, 1972* (ed R.M. West) 32-39 (Department of Biology, Adelphi University, 1972).
48 Gauthier, J. A. Fossil xenosaurid and anguid lizards from the early Eocene Wasatch Formation, southeast Wyoming, and a revision of the Anguioidea. *Contributions to Geology, University of Wyoming* **21**, 7-54 (1982).
49 Hawk, J. R. *Petrology of Eocene carbonates in the Wasatch Formation, southwestern Wyoming*, M.Sc. thesis, San Jose State University, (2001).
50 Smith, K. T. & Gauthier, J. A. Early Eocene lizards of the Wasatch Formation near Bitter Creek, Wyoming: diversity and paleoenvironment during an interval of global warming. *Bull. Peabody Mus. Nat. Hist.* **54**, 135-230 (2013).
51 Stevens, C. E. *Comparative Physiology of the Vertebrate Digestive System*. (Cambridge University Press, 1988).
52 Roberts, N. B. & Taylor, W. H. Comparative pepstatin inhibition studies on individual human pepsins and pepsinogens 1,3 and 5(gastricsin) and pig pepsin A. *J. Enzyme Inhib. Med. Chem.* **18**, 209-217 (2003).
53 Taylor, W. H. Studies on gastric proteolysis; 1. The proteolytic activity of human gastric juice and pig and calf gastric mucosal extracts below pH 5. *Biochem. J.* **71**, 73-83 (1959).
54 Withers, P. C. *Comparative Animal Physiology*. (Saunders College Publishing, 1992).

55 Smeets-Peeters, M., Watson, T., Minekus, M. & Havenaar, R. A review of the physiology of the canine digestive tract related to the development of *in vitro* systems. *Nutr. Res. Rev.* **11**, 45-69 (1998).
56 Sparkes, A. H., Papasouliotis, K., Barr, F. J. & Gruffydd-Jones, T. J. Reference ranges for gastrointestinal transit of barium-impregnated polyethylene spheres in healthy cats. *J. Small Anim. Pract.* **38**, 340-343 (1997).
57 Witmer, L. M. in *Functional Morphology in Vertebrate Paleontology* (ed J.J. Thomason) 19-33 (Cambridge University Press, 1995).
58 Flynn, J. J., Finarelli, J. A., Zehr, S., Hsu, J. & Nedbal, M. A. Molecular phylogeny of the Carnivora (Mammalia): assessing the impact of increased sampling on resolving enigmatic relationships. *Syst. Biol.* **54**, 317-337 (2005).
59 Houston, D. C. & Duke, G. E. in *Raptor Research and Management Techniques* (eds K. L. Bildstein & D. M. Bird) 267-277 (Hancock House, 2007).
60 Duke, G. E., Evanson, O. A. & Jegers, A. A. Meal to pellet intervals in 14 species of captive raptors. *Comparative Biochemistry and Physiology* **53A**, 1-6 (1976).
61 Fuller, M. R. & Duke, G. E. Regulation of pellet egestion: The effects of multiple feedings on meal to pellet intervals in Great Horned Owls. *Comparative Biochemistry and Physiology* **62A**, 439-444 (1979).
62 Fuller, M. R., Duke, G. E. & Eskedahl, D. L. Regulation of pellet egestion: The influence of feeding time and soundproof conditions on meal to pellet intervals of Red-Tailed Hawks. *Comparative Biochemistry and Physiology* **62A**, 433-438 (1979).
63 Farner, D. S. in *Biology and Comparative Physiology of Birds, Volume I* (ed A. J. Marshall) 411-467 (Academic Press, 1960).
64 Reed, C. I. & Reed, B. P. The mechanism of pellet formation in the Great Horned Owl (*Bubo virginianus*). *Science* **68**, 359-360 (1928).
65 Fernández-Jalvo, Y. & Andrews, P. Small mammal taphonomy of Gran Dolina, Atapuerca (Burgos), Spain. *Journal of Archaeological Science* **19**, 407-428 (1992).
